# Impact of Percoll purification on isolation of primary human hepatocytes

**DOI:** 10.1038/s41598-019-43042-8

**Published:** 2019-04-25

**Authors:** R. Horner, J. G. M. V. Gassner, M. Kluge, P. Tang, S. Lippert, K. H. Hillebrandt, S. Moosburner, A. Reutzel-Selke, J. Pratschke, I. M. Sauer, N. Raschzok

**Affiliations:** 1 Charité – Universitätsmedizin Berlin, corporate member of Freie Universität Berlin, Humboldt-Universität zu Berlin, and Berlin Institute of Health, Department of Surgery, Experimental Surgery, Campus Charité Mitte | Campus Virchow-Klinikum, Berlin, Germany; 2 BIH Charité Clinician Scientist Program, Berlin Institute of Health (BIH), Berlin, Germany

**Keywords:** Biomaterials - cells, Hepatocytes, Preclinical research, Translational research

## Abstract

Research and therapeutic applications create a high demand for primary human hepatocytes. The limiting factor for their utilization is the availability of metabolically active hepatocytes in large quantities. Centrifugation through Percoll, which is commonly performed during hepatocyte isolation, has so far not been systematically evaluated in the scientific literature. 27 hepatocyte isolations were performed using a two-step perfusion technique on tissue obtained from partial liver resections. Cells were seeded with or without having undergone the centrifugation step through 25% Percoll. Cell yield, function, purity, viability and rate of bacterial contamination were assessed over a period of 6 days. Viable yield without Percoll purification was 42.4 × 10^6^ (SEM ± 4.6 × 10^6^) cells/g tissue. An average of 59% of cells were recovered after Percoll treatment. There were neither significant differences in the functional performance of cells, nor regarding presence of non-parenchymal liver cells. In five cases with initial viability of <80%, viability was significantly increased by Percoll purification (71.6 to 87.7%, p = 0.03). Considering our data and the massive cell loss due to Percoll purification, we suggest that this step can be omitted if the initial viability is high, whereas low viabilities can be improved by Percoll centrifugation.

## Introduction

Primary human hepatocytes serve as the gold-standard model for *in vitro* testing of drugs that are metabolized in the liver^1,2^. In order to address the 3R-principle^3^, animal testing could be reduced with hepatocytes available on a grand scale. With highly metabolically active *in vitro* 3D-cultures of primary human hepatocytes, pharmacokinetic processes of drug metabolism can be investigated without the need for using a xenogenous animal model^4,5^. Primary human hepatocytes can be used to study the pathogenesis of human liver diseases more thoroughly, especially in regard to the increasing number of patients suffering from non-alcoholic fatty liver disease^6^. Moreover, in face of the growing organ scarcity for liver transplantation, hepatocytes can help to provide bridging strategies until a suitable donor organ is available, e.g. by hepatocyte transplantation^7–9^, or be used for bioengineering of neo-organs^10,11^. The wide application of primary human hepatocytes is, however, limited due to the lack of a safe, constant source of high-quality human liver cells.

**Figure 1:**
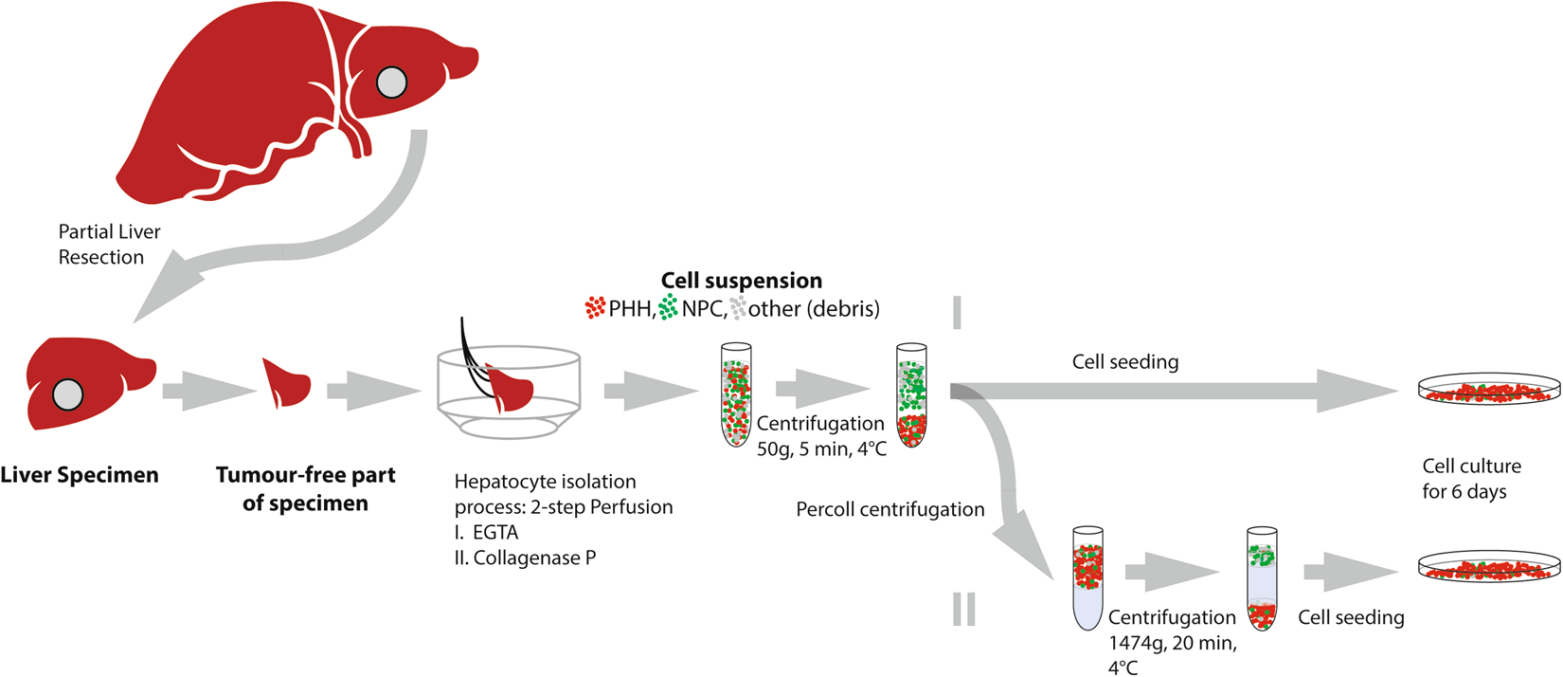
Tissue retrieval and study design. Abbreviations: PHH: Primary human hepatocytes, NPC: non-parenchymal cells, EGTA: ethylene glycol tetraacetic acid.

Collagenase perfusion protocols have been an established procedure since the 1980s for isolation of primary human hepatocytes^12^. The best available and most commonly used source for human liver cells is tissue obtained from partial liver resection for therapeutic purposes, for example after removing a tumor (Fig. 1). After cannulation, perfusion and enzymatic digestion of the tissue specimen, the resulting cell suspension contains liver cells that can be cultured for subsequent use. Optionally, hepatocyte suspensions are purified through density separation with Percoll, which is a low-density fluid containing colloidal silica particles coated with polyvinylpyrrolidone^13,14^. This additional purification step is included to the isolation protocol in almost 50% of published studies on primary human hepatocytes in the literature reviewed for this study (see supplementary data). Percoll purification can be used for splitting up different liver cell populations, for example Kupffer cells, stellate cells, hepatic progenitor cells, oval cells or liver sinusoidal endothelial cells by centrifuging the cell suspension through differently concentrated layers of Percoll^15–18^. Moreover, several publications^19–21^ report a Percoll purification step in their protocol to recover liver cells after cryopreservation. Nonetheless, density purification is also commonly performed to purify parenchymal human hepatocytes themselves^22–26^, adding about 45 minutes to the 3 hours isolation procedure. Systemic evaluation of this cell- and time-consuming step in hepatocyte isolation is not currently available in the literature, specifically not for human primary liver cells.

With respect to the fact that the wider use of primary human hepatocytes is mainly limited by the lack of sufficient amount of hepatocytes^11,27^, it is crucial to know whether or not the Percoll purification step is necessary at all during hepatocyte isolation. The aim of this work is to investigate whether purification of primary human hepatocytes with density separation by Percoll has a beneficial effect on the performance of isolated human hepatocytes; e. g. yield, viability, plating efficiency, metabolic function, purity, bacterial contamination levels^28^ and purity of cell cultures.

## Methods

### Study design and tissue retrieval

From October 2015 to July 2016, a total of 27 hepatocyte isolations were performed at the Department of Surgery, Campus Charité Mitte | Campus Virchow-Klinikum of the Charité – Universitätsmedizin Berlin (Germany). Liver tissue specimens were retrieved from patients undergoing partial hepatectomies. Informed consent was obtained from all tissue donors, and all institutional and ethical guidelines were followed (approval of local ethics committee “Ethikkommission der Charité – Universitätsmedizin Berlin”: EA2/137/09). Patients with infectious diseases such as viral hepatitis, multi-resistant bacteria, or echinococcosis were excluded from the study. All donor-related information was collected from the patient’s hospital records.

Liver tissue was examined for macroscopically tumor-free areas immediately after resection in the operating room. Tissue specimens used for hepatocyte isolation were cut with a scalpel with only one dissection surface, the rest of the liver specimen being covered by Glisson’s capsule. Mass of these specimens was 18.21 g on average (SEM ± 2.64 g). Tissue specimens were stored in a container filled with 4 °C cold Williams E-medium and transferred to the laboratory. All steps were performed under sterile conditions and the isolation protocol began within 30 minutes after retrieval of the specimen (=cold ischemic time). To prevent interpersonal variability, all hepatocyte isolations were carried out by a single person (RH).

### Hepatocyte isolation and culturing

Hepatocytes were isolated using an established two-step-perfusion protocol with Collagenase P^29^. For this technique, sectioned major vessels were cannulated with 3–5 cannulas (custum-made stainless-stell irrigation cannulas) that where connected to thin plastic tubes, merged in a larger tube, and then connected to a roller pump for perfusion. Cannulas and major leakages were sealed with a histoacryl-tissue-glue (3 M, St. Paul, MN, USA). First, the tissue was rinsed with pre-warmed (37 °C) perfusion solution (containing 1.42 M NaCl, 67 mM KCl, and 100 mM 4-(2-hydroxyethyl)-1-piperazineethanesulfonic acid [all chemicals from Carl Roth GmbH, Karlsruhe, Germany]) supplemented with ethylene glycol tetraacetic acid (EGTA, Serva, Heidelberg, Germany) which is a chelator for Calcium ions that facilitates flushing the blood out of the specimen, hinder blood from clotting as well as disrupting the desmosomes between cells. This step was performed for 11.06 minutes (SEM ± 0.47) on average. Secondly, the liver specimen was perfused with recirculating perfusion solution containing 1 mg collagenase P/mL (Roche Diagnostics GmbH, Mannheim, Germany) at 37 °C. Digestion was stopped when the tissue was visually and tactically deemed digested with a mean digestion time of 8.07 minutes (SEM ± 0.24). Afterwards, the specimen was transferred into a petri dish containing perfusion solution without EGTA but supplemented with 4% human albumin to stop the enzymatic digestion. After removal of the cannulas, tissue was disrupted mechanically by shaking and using tweezers to disrupt cells from the remaining scaffold structures.

The resulting cell suspension was then centrifuged (5 minutes, 50 g, 4 °C) to eliminate cell debris. After resuspending in culture medium (phenol red free Williams E with supplements: 1 µM Insulin [Lilly, Indianapolis], 1 µM Fortecortin [Merck Serono GmbH, Darmstadt, Germany], 1 mM sodium pyruvate, 10 mM HEPES-Buffer and 10% fetal calf serum [all from Biochrom]), cells were counted, and viability was assessed using the trypan blue exclusion test. A predetermined part of this “crude” suspension, i.e. without having undergone the Percoll purification step, was then seeded at a density of 1041 cells/mm² (1 million cells/well of a 6-well-plate) onto collagen-coated culture plates (Biochrom GmbH, Berlin, Germany) for cultivation.

Remaining cell suspension underwent the Percoll density separation by centrifugation through a Percoll layer (25% Percoll [Biochrom GmbH, Berlin, Germany] in phosphate buffered saline, 1.037 g/ml, 300mosm) for 20 minutes at 1474 g (4 °C). Therefore, 5 mL of cell suspension was gently mounted over a cushion of 15 mL Percoll-suspension. After careful aspiration of the supernatant, cell pellets were re-suspended with ice-cold PBS and centrifuged (5 minutes, 50 g, 4 °C) to wash out Percoll particles. After resuspension in medium, cells were counted and seeded as described above (Fig. 1). The cells were washed with pre-warmed PBS 4 hours after seeding to discard non-attaching cells. Liver cells were cultured in modified Williams E (see above, additional 100 U/mL/100 µg penicillin/streptomycin [Biochrom GmbH, Berlin, Germany]) for 6 days at 37 °C, 21% O_2_, 5% CO_2_. Medium was changed every 24 hours during the cultivation period.

### Measurement of cell culture parameters

To measure the aspartate aminotransferase (AST) activity, albumin production and urea content, the supernatant of 3 pooled wells was centrifuged (3000 g, 5 min, 4 °C) after culturing overnight and after 2, 4, and 6 days in culture. Measurement of AST and Urea was conducted by Labor Berlin – Charité Vivantes GmbH (Berlin, Germany) within 12 hours of cold storage of the samples using an enzymatic assay (Roche Hitachi cobas c 6000 system, Roche Diagnostics GmbH, Mannheim, Germany). Albumin production was assessed using the human albumin ELISA kit (Bethyl Laboratories, Montgomery, TX, USA) after supernatant sample storage at −80 °C. ELISA samples were measured as duplets and according to the manufacturer’s instructions. All data was set in relation to the protein-content of the respective wells at the end of culture (day 6). For protein content measurement, the cells from these wells were washed, scratched off the culture plates, pooled, suspended with RIPA-Buffer and stored at −80 °C until being measured. Finally, photometric analysis using BCA-Reagent (Pierce, Thermo Fisher Scientific, MA, USA) according to the manufacturers’ instructions was performed with all samples measured in triplets. Plating efficiency was evaluated using a protocol as described elsewhere^29^: The cells were washed 24 hours after seeding them. Subsequently, the adherent protein was set in relation to the sum of the adherent protein and the total protein contained in the supernatants at that time-point.

### Purity assay

Purity of the composition of the cell culture was assessed using real-time quantitative (q-rt)-PCR. RNA samples were collected from 12 isolations 4 h after seeding, on days 2, 4 and 6 of culture. After washing with PBS, cells were pooled in RNAase-free Eppendorfer containers containing TRIzol^®^ (Thermo Fisher Scientific, Waltham, MA, USA) and immediately shock frozen in liquid nitrogen. Isolation and RNA measurements were performed according to the established protocols using a NanoDrop 2000c UV-Vis spectrophotometer (Thermo Fisher Scientific, Waltham, MA, USA) for calculating RNA-concentration. The RevertAid First Strand cDNA Synthesis Kit (Thermo Fisher Scientific, Waltham, MA, USA) in a PTC-100® Thermal Cycler (Bio-Rad Laboratories, Inc., Hercules, CA, USA) was used for generating cDNA. Gene expression was assessed using a StepOne™ RT-PCR cycler (ThermoFisher Scientific, Venlo, Netherlands). The following established DNA-primers were used (Abcam, Cambridge, United Kingdom): KRT18 (hepatocytes, Vim (fibroblasts), GGT1 (cholangiocytes), Pecam1 (sinusoidal endothelial cells) and CD68 (Kupffer cells). B2M was chosen as an endogenous control because it had been established to be unregulated in random samples before. Results are shown using the R = 2^(−ΔΔCT)^ method as described elsewhere^30^.

Additionally, these markers were stained in an immunofluorescence assay on culture plates that were fixed 24 h after seeding and at the end of the culture period. Cells were fixated with 4% formaldehyde and stored in methanol until staining.

### Microbiological testing

In 16 isolations, cell suspension underwent microbiological testing directly before seeding. A fraction of 5 ml of the cell suspension were transferred into aerobic and anaerobic blood culture bottles before and after Percoll purification. During 5 days of incubation, bacterial growth was examined by the Labor Berlin – Charité Vivantes GmbH (Berlin, Germany).

### Statistical analysis

Statistical analysis was performed using GraphPad Prism 6.0 (GraphPad Software, Inc., La Jolla, CA, USA). After testing Gaussian distribution with the Shapiro-Wilk-test, either a paired t-test or a Wilcoxon matched-pairs-signed-rank-test was done to analyze paired quantitative parameters. A p-value less than 0.05 was considered significant. All data is expressed as the mean ± standard error of the mean (SEM).

## Results

### Isolation outcome

**Figure 2:**
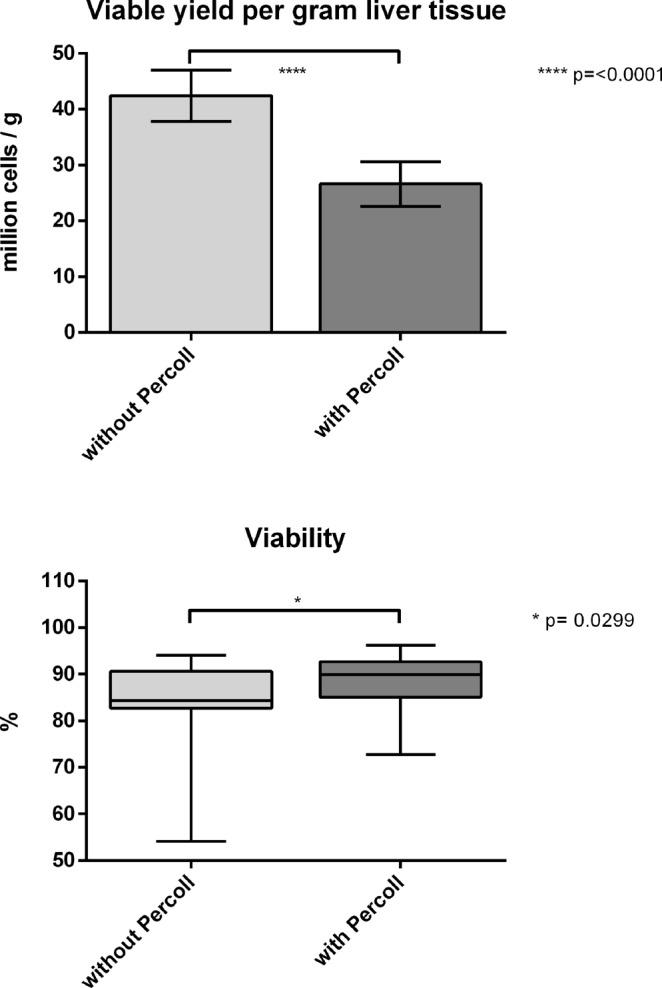
Yield and viability of the overall group. Comparison of liver cell isolation without vs. with Percoll purification, n = 27. Viability: Box plots show the 25th and 75th percentile and median value, whiskers display smallest and largest value. Yield: Columns display mean value, whiskers indicate SEM.

Isolation outcome parameters are expressed in relation to the respective mass of the liver specimen. The isolation outcome yielded 42.4 (±4.6) × 10^6^ isolated cells per gram liver tissue on crude cells without Percoll purification and 26.6 (±4.0) × 10^6^ cells per gram liver tissue after Percoll purification (Fig. 2). This resulted in an average Percoll survival rate of 59.03% (±3.9). Viability was significantly increased (p = 0.0299) from 84.47% (±1.60) to 88.18% (±1.13) by Percoll purification.

### Functional parameters

**Figure 3:**
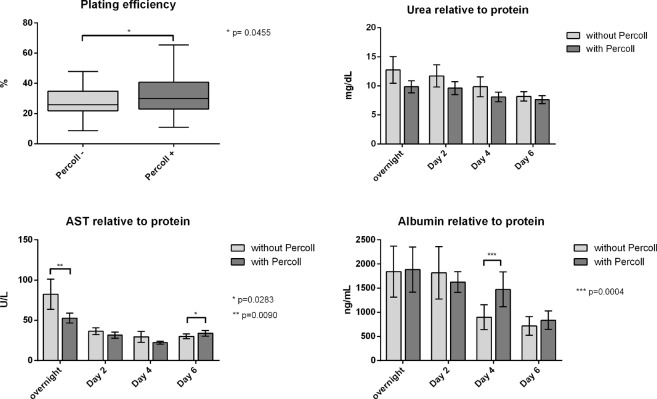
Functional parameters of the overall group. Comparison of hepatocytes isolated without or with Percoll purification (n = 27). Plating efficiency: Box plots show the 25^th^ and 75^th^ percentile and median value, whiskers display smallest and largest value. Urea, AST, Albumin: Columns display mean value, whiskers indicate SEM. Abbreviations: AST: aspartate aminotransferase, SEM: standard error of mean.

Comparing functional parameters of isolated cells, plating efficiency was significantly (p = 0.046) increased in Percoll-purified cells (from 27.19 ± 2.19% to 31.77 ± 2.98%). Albumin, urea and AST levels are shown relative to the respective protein content at the end of culture (Table 1, Fig. 3).

**Table 1:** *In vitro* hepatocyte function.

Parameter	Overnight			Day 2			Day 4			Day 6		
	Mean ± SEM		p-Value	Mean ± SEM		p-Value	Mean ± SEM		p-Value	Mean ± SEM		p-Value
	Percoll−	Percoll+		Percoll−	Percoll+		Percoll−	Percoll+		Percoll−	Percoll+	
Urea (mg/dL)	12.74 ± 2.30	9.84 ± 1.03	0.0532	11.71 ± 1.89	9.61 ± 1.12	0.2722	9.83 ± 1.70	8.10 ± 0.83	0.3205	8.18 ± 0.81	7.63 ± 0.69	0.1111
Albumin (ng/mL)	1843.41 ± 529.30	1884.05 ± 466.00	0.8906	1817.28 ± 542.40	1625.02 ± 215.40	0.3683	897.36 ± 259.00	1474.38 ± 363.20	**0**.**0004**	716.08 ± 192.82	835.26 ± 191.45	0.3225
AST (U/L)	82.37 ± 18.67	52.71 ± 6.13	**0**.**0090**	36.56 ± 4.27	31.56 ± 3.83	0.0516	29.38 ± 6.67	22.14 ± 1.96	0.3525	29.98 ± 2.96	33.77 ± 3.62	**0**.**0425**

Urea levels were similar in both groups throughout the cultivation period, being slightly higher in the untreated group, however without reaching statistical significance. Aspartate aminotransferase release was initially higher in the untreated group (82.37 ± 18.67 U/L vs. 52.71 ± 6.129 U/L, p = 0.009), whereas towards the end of culture Percoll-purified cells showed higher levels (29.98 ± 2.959 U/L vs 33.77 ± 3.622 U/L, p = 0.028). Albumin content decreases in both groups over the course of cell culture, however, additionally purified cells tended to decrease their production later and to a lesser degree than cells without Percoll purification. This reached statistical significance (p = 0.0004) on day 4: while Albumin content was 897.36 ng/mL (±259) without Percoll treatment, levels were higher on Percoll purified cells 1474.38 ng/mL (±363.2).

### Microbiological analysis

Microbiological analysis revealed no difference in contamination rates between the two groups. Of the 16 isolations that underwent this investigation, 13 were sterile while three were contaminated. Identified pathogens were *Enterococcus faecium*, *E*. *faecalis* and *E*. *coli* as well as *Klebsiella oxytoca* and *Staphylococcus haemolyticus*. However, there was no difference between the two groups and we presume that this contamination most likely occurred in the operating room during retrieval of the tissue.

### Cell culture purity

**Figure 4:**
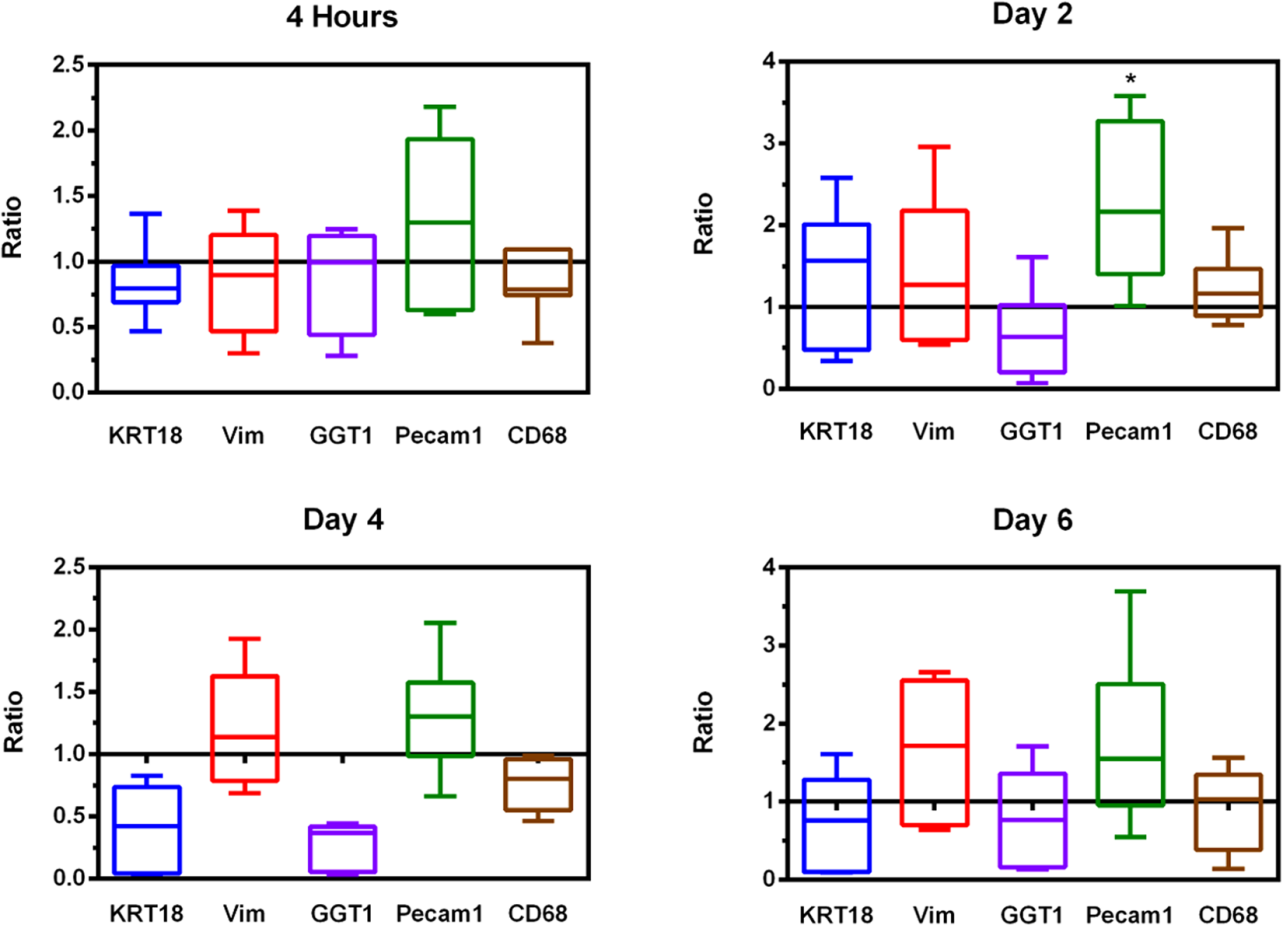
q-rt-PCR. q-rt-PCR displayed by the 2^−ΔΔCT^-Method: values > 1 indicate higher expression of the gene in preparations without Percoll purification, while values < 1 mean higher expression in the Percoll-purified samples. Box plots show the 25^th^ and 75^th^ percentile and median value, whiskers display smallest and largest value.

Purity assays showed no difference in the expression of liver-cell-specific DNA. KRT18 levels as well as CD68 on Kupffer cells and Vimentin on fibroblasts were the same in both the crude as well as the Percoll-purified liver cell cultures. However, there was a slight tendency in crude, i.e. non Percoll-purified, preparations to express more Pecam1, which is a marker for sinusoidal endothelial cells. This reached statistical significance on Day 2 of culturing (2.12-fold higher expression in crude cultures, p = 0.0220). Moreover, the presence of GGT1-expressing cells, namely cholangiocytes, tends to be higher in preparations that have undergone Percoll purification. This trend is visible throughout the culture period, and nearly reaches statistical significance on day 4, when expression is 5.66-fold higher in the untreated group (p = 0.0616, Fig. 4).

**Figure 5:**
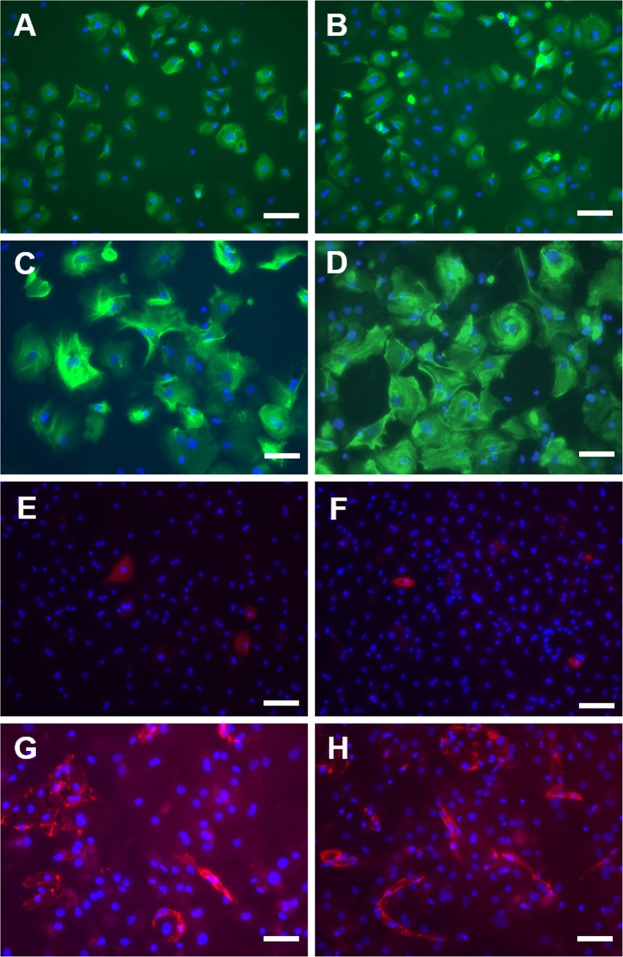
Immunofluorescence. Fluorescence microscopy of hepatocyte cultures without (**A**,**C**,**E**,**G**) of after (**B**,**D**,**F**,**H**) purification with Percoll. Time points: 24 hours after seeding (**A**,**B**,**E**,**F**) and on Day 6 (**C**,**D**,**G**,**H**). A-D display KRT18-staining (hepatocytes), E-H display Pecam1-staining (endothelial cells). Scale bar: 50 µm.

**Figure 6:**
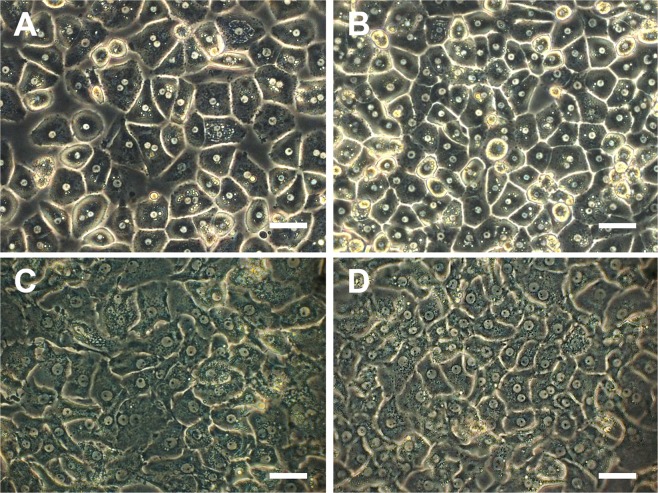
Phase contrast images. Phase contrast microscopy of hepatocyte cultures without (**A**,**C**) of after (**B**,**D**) purification with Percoll. Time points: 24 hours after seeding (**A**,**B**) and on Day 6 (**C**,**D**). Scale bar: 50 µm.

Immunofluorescence staining showed no differences between the two groups and generally supports the presence of all hepatic cell types in both groups, detected by mRNA-expression profiles. While hepatocytes in the KRT-18-staining look compact 24 hours after seeding, they appear protruded and not as dense as at the beginning of culture. This is true for both crude and Percoll-treated preparations. Interestingly, while Pecam-1-postive cells, i.e. endothelial cells, are also compact 24 hours after seeding, they form networks at the end of the culturing period (Fig. 5). There were again no differences detectable between the two treatment groups. Fractions of non-parenchymal cells were also stained, however due to difficult quantification not showed in this publication. Stellate cells were not found in either group. See supplementary information material for the immunofluorescence staining images of different non-parenchymal cell populations. Phase contrast microscopy of the cultures 24 hours after seeding and at the end of culture revealed no differences between the two treatment groups (Fig. 6).

### Subgroup analysis

When analyzing the data more thoroughly, five cases presented an initial viability that was lower than 80%. Percoll purification increased the viability from 71.63 (±4.44) to 87.7% (±2.69) in these isolations, reaching statistical significance p = 0.0299. However, no benefit in functional parameters could be detected. An average of 56.4% of the cells could be recovered from Percoll-purification, whereas yield was significantly diminished from 25.90 (±9.72) × 10^6^ to 15.3 (±8.66) × 10^6^ cells per gram of liver tissue (Table 2). When analyzing the features of this subgroup, the three cases differed from the overall group in a few known factors^24,31–33^ that are known to affect the isolation outcome, including preoperative levels of ALT, AST, AP, GGT and in the operating technique (Table 3).

**Table 2:** Subgroup analysis of isolation outcome parameters.

Parameter	Overall			high initial viability > 80%			low initial viability <80%		
	Mean ± SEM		p-Value	Mean ± SEM		p-Value	Mean ± SEM		p-Value
	Percoll−	Percoll+		Percoll−	Percoll+		Percoll−	Percoll+	
Plating efficiency (%)	27.19 ± 2.19	31.77 ± 2.99	**0**.**0455**	29.92 ± 1.87	34.33 ± 3.05	0.0783	11.735 ± 2.47	16.46 ± 3.48	0.4312
Viablility (%)	84.47 ± 1.60	88.18 ± 1.13	**0**.**0299**	87.39 ± 0.91	88.29 ± 1.27	0.3209	71.63 ± 4.44	87.70 ± 2.79	**0**.**0625**
Percoll survival (%)	**59**.**03** ± **3**.**90**			**59**.**63** ± **4**.**10**			**56**.**38** ± **12**.**02**		
Relative yield in millions per gram liver tissue	42.40 ± 4.60	26.60 ± 4.00	<**0**.**0001**	46.15 ± 4.95	29.18 ± 4.40	<**0**.**0001**	25.90 ± 9.72	15.30 ± 8.66	**0**.**0299**
Total protein on culture plate on day 6	596.20 ± 66.79	662.70 ± 61.98	**0**.**0102**	643.76 ± 73.55	716.71 ± 66.50	**0**.**0156**	334.38 ± 79.79	365.58 ± 55.33	0.6250

**Table 3:** Subgroup features.

Parameter	High initial viability >80%	Low initial viability <80%
	Mean ± SEM	Mean ± SEM
**Number of cases**	22	5
**Surgical Indication**		
HCC	2	0
Biliary tree carcinoma	6	4
CRLM	12	0
Benign disease	2	1
**Preoperative bilirubin**	0.65 ± 0.08	1.21 ± 0.49
**Preoperative ALT**	35.32 ± 4.98	63.00 ± 19.99
**Preoperative AST**	41.24 ± 6.10	45.60 ± 4.41
**Preoperative AP**	152.50 ± 41.62	320.60 ± 108.62
**Preoperative GGT**	215.05 ± 59.80	356.80 ± 133.79
**Preoperative INR**	1.02 ± 0.01	1.04 ± 0.02
**Age of donor (years)**	61.50 ± 3.13	57.40 ± 7.69
**Fibrosis**		
No Fibrosis	5	0
Minimal (periportal)	6	2
Mild (septs)	4	3
Severe Fibrosis (portocentral septs, architecture changes)	2	0
Cirrhosis (definite architectural changes)	1	0
Data not available	4	0
**Steatosis**		
No Steatosis	2	0
≤30% Steatosis	8	0
>30% Steatosis	3	0
Data not available	9	5
**Cold ischeamic time (min)**	23.27 ± 3.51	15.00 ± 2.24
**Time of first perfusion (min)**	11.18 ± 0.56	10.50 ± 0.61
**Time of second perfusion (min)**	8.09 ± 0.28	8.00 ± 0.57
**Mass of liver specimen (g)**	16.34 ± 1.39	26.42 ± 13.42

Abbreviations: GGT, Gamma-glutamyl transpeptidase; CIT, cold ischemic time; AST, aspartate aminotransferase; ALT, alanine aminotransferase; AP, alkaline phosphatase; INR, international normalized ratio; HCC, Hepatocellular carcinoma; CRLM, Colorectal liver metastasis.

## Discussion

Isolation of primary human hepatocytes is an expensive and time-demanding process. It requires a high-level hepatobiliary surgical center, excellent communication between the personnel in the operation room to keep ischemic times as short as possible and experienced laboratory staff, as well as a well-equipped laboratory infrastructure. Tissue specimens retrieved from the operating room for hepatocyte isolation must therefore be utilized as effectively as possible.

Using Percoll density centrifugation is a double-edged sword: Is the tremendous loss of cells worth the better performance? In all publications that provide detailed information regarding the Percoll purification step during isolation of human liver cells^22,23,33–36^, the cell yield was diminished by this procedure, while the viability improved. According to Schröder *et al*.^23^ who performed purification through a 50% concentrated Percoll-suspension, a very pure cell culture was retrieved with few contaminating non-parenchymal liver cells (assessed using immunohistological staining) and a viability of 94%. However, in seven of 32 experiments no cells were recovered after the Percoll purification step, while a low-yield after Percoll purification was reported in the remaining 25 trials. Olinga *et al*.^22^ declared an average recovery threshold of 33% after Percoll purification, but viability improved from 69% to 92%. Information on the quality and purity of the resulting cells was not reported though. Laba *et al*.^34^ centrifuged their cell suspensions through a 29–31%-concentrated Percoll layer before and after cryopreservation. Mean viability of crude cells before Percoll purification ranged up to 72% and improved to 89% after Percoll purification. When comparing freshly isolated crude cell suspensions to cryopreserved cells that were purified using the Percoll method after thawing, a positive effect on the plating efficiency was found after usage of Percoll (35–40% improved to 65–85%) and lower levels of non-parenchymal cells were reported. However, this study focused on the comparison of fresh and cryopreserved and thawed hepatocytes, while there was no metabolic test and the study was limited due to the low number of experiments (n = 5). These authors also did not directly compare cells before and after Percoll purification regarding cell culture purity and plating efficiency, which are determining parameters for the subsequent application of hepatocytes:

Primary human hepatocytes are widely used to study pharmacodynamic processes as well as for investigating processes of the hepatocyte itself. Apart from *in vitro* research, human hepatocytes can also be applied in the clinic. Since the number of donated organs for whole organ transplantation is limited, but the number of patients with end-stage liver diseases is rising^37^ the demand for an alternative to whole orthotropic liver transplantation is high. Hepatocytes can be used to bridge the time until a suitable donor organ is available^9^. Hepatocyte transplantation has been successfully performed in children with inherited metabolic diseases such as Crigler-Najjar syndrome^7,8^ and in mice with hemophilia^38^. Primary hepatocytes can also be cultured in spheres or in multi-layered^27^ cell sheet technology. These three-dimensional constructs serve as a cell-based regenerative therapy by providing metabolic function when implanted in the kidney capsules of mice. Moreover, bioengineered organs are under investigation to overcome the shortage of donor organs for example by using human hepatocytes to engraft matrices derived from decellularized livers^10,39,40^. Tissue engineered organs still face problems of sufficient re-endothelialization, or reperfusion injury due to inflammatory mediators secreted by Kupffer cells contaminating the hepatocyte suspension^41^. Therefore, providing hepatocyte cultures in high purity is necessary. In contrary to ours and others expectations, our data show that Percoll purification at 25% concentration significantly contributes towards a more homogenously pure cell culture. Higher concentrated Percoll cushions (e.g. 50%) could achieve this. However, with increased silica particle density then fatty – but metabolically active – hepatocytes would be sorted out and lost. Hence, ideally, Percoll concentrations would be adjusted to the fat content of the donor’s liver. However, such an adjustment of the cell isolation procedure is not practical as often no suitable histopathological assessment is available prior to the isolation process. Although the overall metabolic performance of cells is similar in both treatment groups, AST levels differed significantly 24 hours and at the end of culture. We hypothesize that the difference on day 1 is due to the fact that there is more cell debris and dead cells in the non-Percoll-purified culture plates. This debris does not adhere for further culture, however, contributes to the high levels of AST in the non-purified group on day 1. The differences between albumin levels on day 4 and AST levels on day 6 might indicate a temporal higher metabolic activity of hepatocytes isolated with Percoll purification which is however not consistent throughout the culture period. Our study is limited by the fact that we analyzed hepatocytes isolations from surgically resected liver tissue, which would most likely not be applicable for therapeutic use. We do, however, expect that we would obtain similar results when comparing liver cell isolations from whole liver grafts with vs. without Percoll purification.

Primary liver cells isolated from human tissue still face the hurdle of availability. The limited preservation of metabolically capable primary hepatocytes *in-vitro* has created an urgent need for an alternative to primary human hepatocytes. Even though genetically engineered hepatic cells are under thorough investigation in stem cell research^42–45^, primary human hepatocytes are still the gold standard for the applications mentioned before^9,27^.

Given the fact that Percoll purification could not raise the function of the cells, we suggest that density separation is only useful to sort out initial dead cells and debris – if needed. If the cell suspension has a high initial viability, then there is no evidence to support the need for Percoll purification. Regarding functional effects of this step, there are no benefits, but also no contradictions to the centrifugation through the silica-particle-layer could be found. If donors do not meet the criteria for ensuring a good hepatocyte isolation outcome, as reported in the literature, then Percoll purification could still be considered to rescue a fraction of highly viably hepatocytes.

## Supplementary information

Supplementary information and data

## Data Availability

The datasets generated during and analysed during the current study are available from the corresponding author on reasonable request.
