# Supplementary material for: Impact of Percoll purification on isolation of primary human hepatocytes
Source: Sci Rep. 2019 Apr 25;9:6542. doi: 10.1038/s41598-019-43042-8 (PMC6484008; doi:10.1038/s41598-019-43042-8)
Supplement: Supplementary file 1 — Supplementary information and data [file 41598_2019_43042_MOESM1_ESM.pdf]

Impact of Percoll purification on isolation of primary human hepatocytes – Supplementary information

Authors:

Horner R (1), Gassner JGMV(1), Kluge M(1), Tang P(1), Lippert S(1), Hillebrandt KH(1), Moosburner S(1), Reutzel-Selke A(1), Pratschke J(1), Sauer IM(1), Raschzok N(1) (2)*

Affiliation:

1) Charité – Universitätsmedizin Berlin, corporate member of Freie Universität Berlin, Humboldt-Universität zu Berlin, and Berlin Institute of Health, Department of Surgery, Experimental Surgery, Campus Charité Mitte | Campus Virchow-Klinikum, Berlin, Germany

2) BIH Charité Clinician Scientist Program, Berlin Institute of Health (BIH), Berlin, Germany

Supplementary information (1)

Immunofluorescence Staining of different liver cell types

Fluorescence microscopy of hepatocyte cultures without (left column) of after (right column) purification with Percoll. Time points: 24 hours after seeding (first row for each staining respectively) and on Day 6 (second row for each staining respectively). A-D display KRT18-staining (hepatocytes), E-H display Pecam1-staining (endothelial cells). Scale bar: 50µm

Without - With Percoll

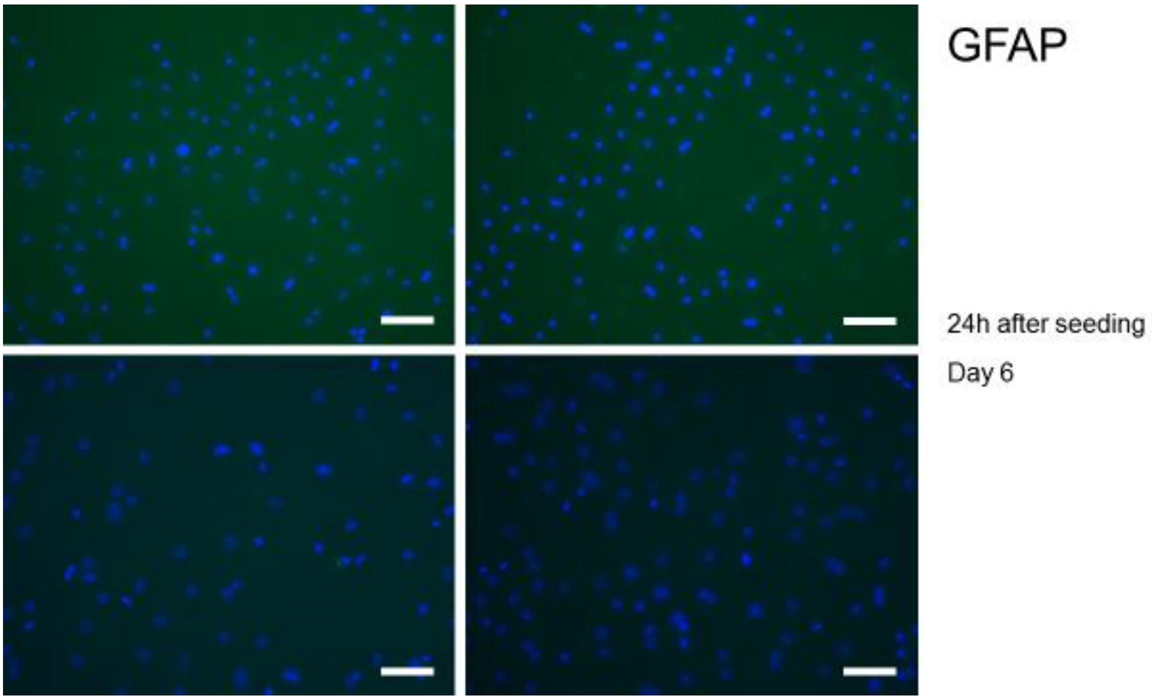

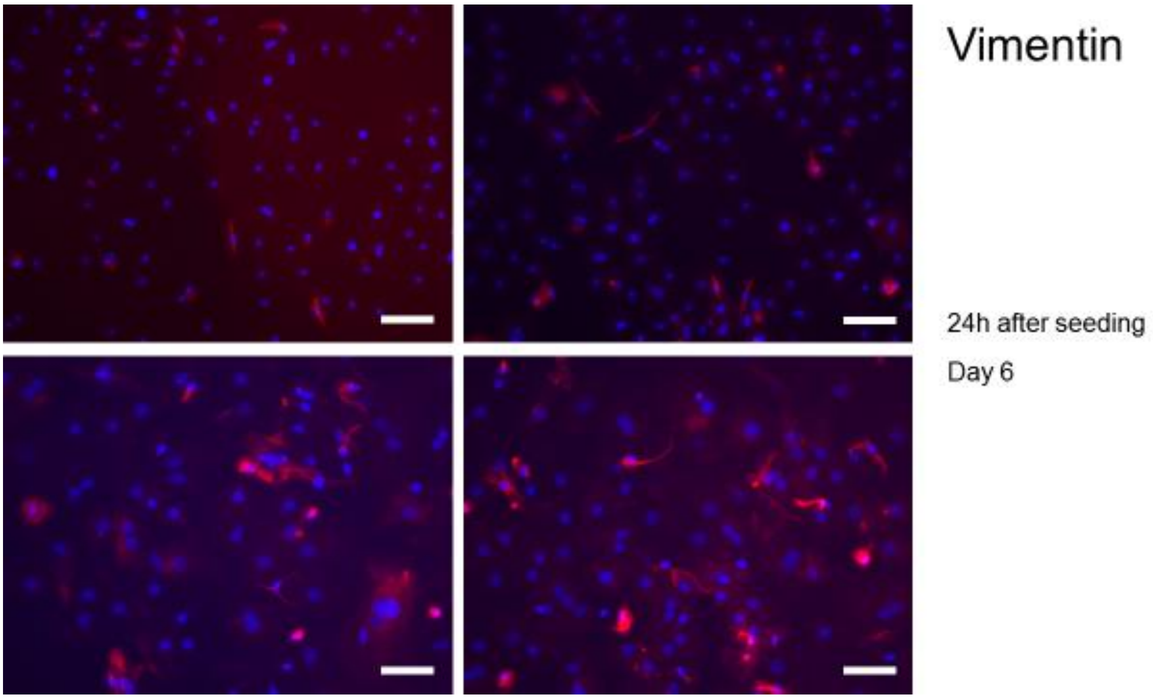

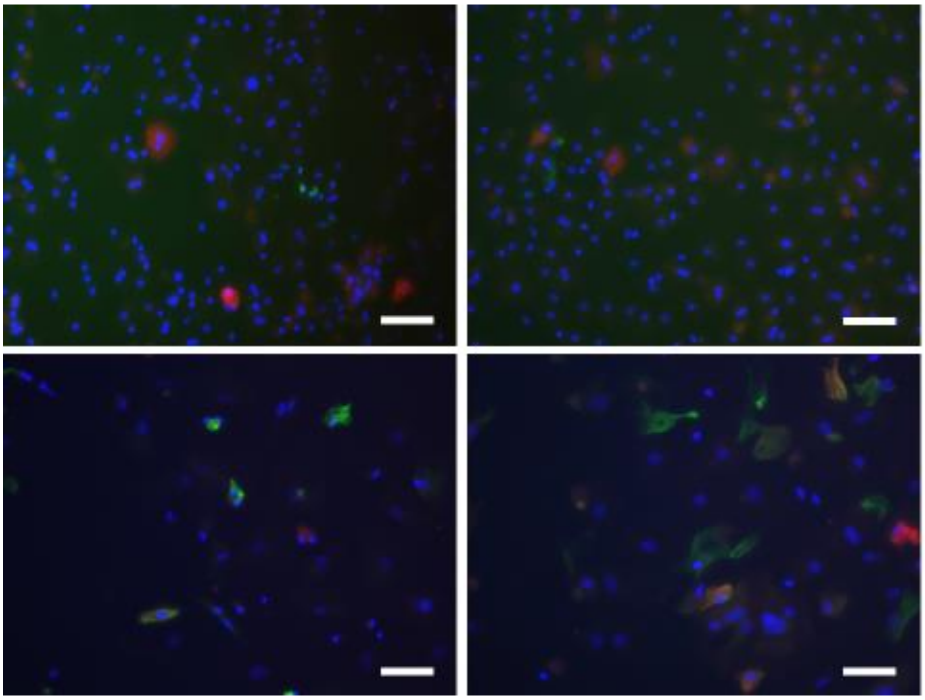

KRT19/
KRT7

24h after seeding

Day 6

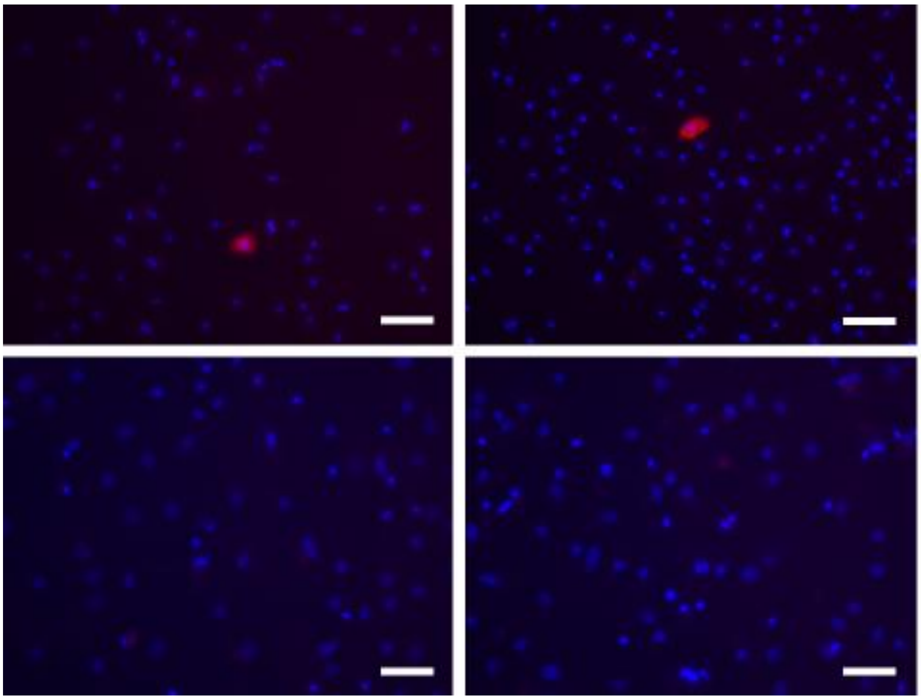

EpCam

24h after seeding

Day 6

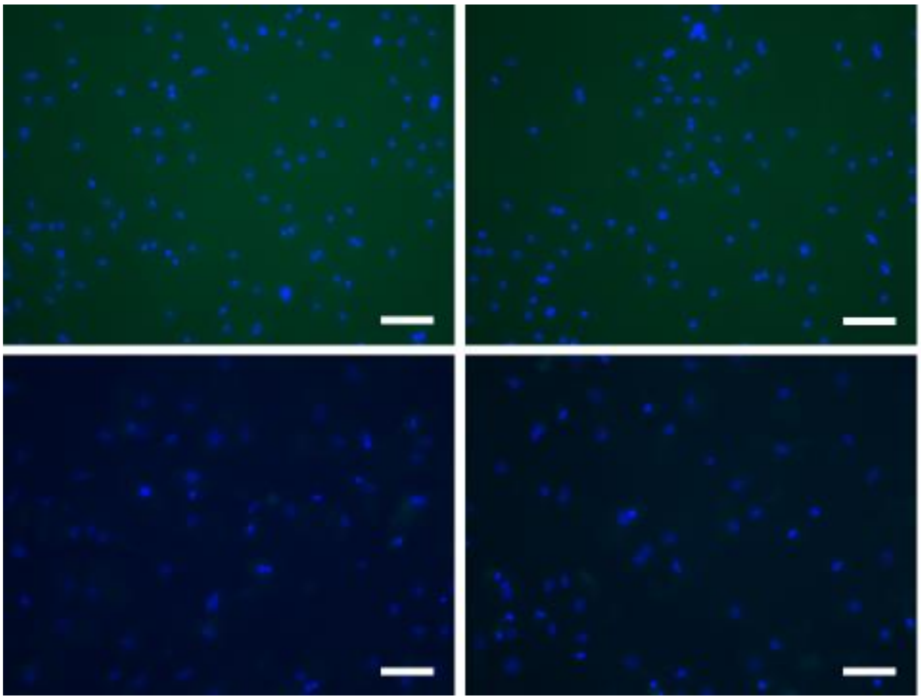

CD68

24h after seeding

Day 6

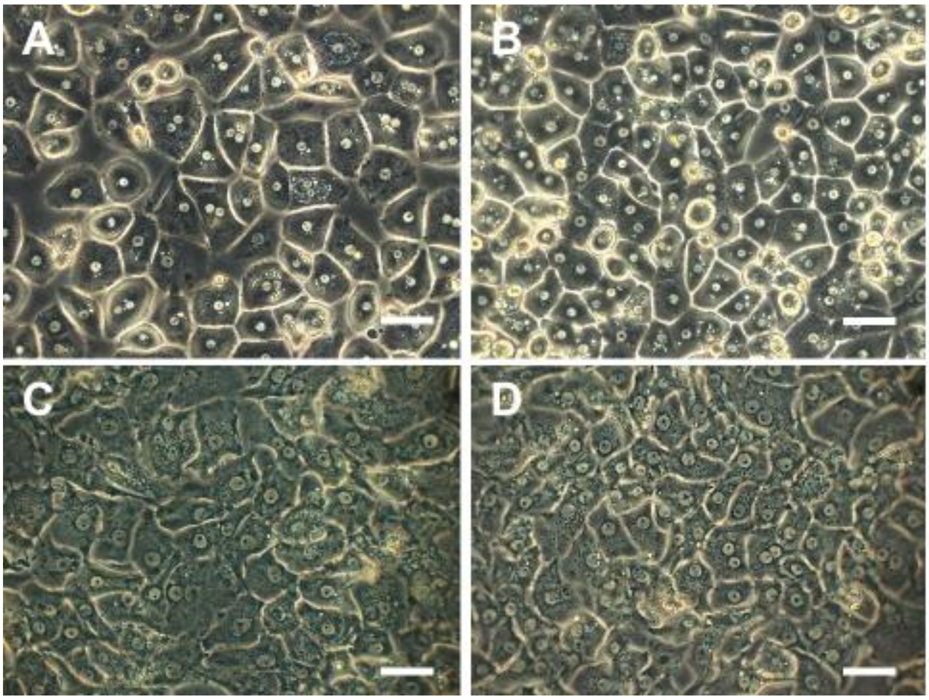

Phasecontrast

24h after seeding

Day 6

Supplementary information (2) – Literature Research - Evaluation of the benefit of Percoll purification in primary human hepatocyte isolation

Search strategy and exclusion criteria

On May 28th, 2017, a systematic literature search was performed in PubMed (MEDLINE) using the following two search terms:

MeshTerm 1:

("Hepatocyte* Isolation" OR "primary hepatocyte*" OR "human hepatocyte*" OR ("Liver" AND "cell isolation")) AND ("plating efficiency" OR “density purification" OR Percoll OR Purification OR "Gradient Centrifugation" OR "density gradient" OR "culture purity")

MeshTerm 2:

"Hepatocyte* isolation"

All papers (n=352) where exported into Endnote X9 (Thomson Reuters, New York, US) and screened for duplicates (n=16). The resulting 326 papers were then screened by reading the title and abstract. After reading the abstracts, 170 papers were excluded because their abstract revealed that they fulfil exclusion criteria, including:

- Paper did not work with primary isolated hepatocytes but with cell lines like HuH7 or HepG2
- Paper focused on pharmaceutical drug-safety testing and gave no detailed information about the hepatocytes they used
- Paper used non-mammalian primary hepatocytes

After removal of papers which met the exclusion criteria, 156 papers were cross read. During this process publications were categorized for second exclusion:

Of all the publications that were identified to deal with primary hepatocytes and meet aforementioned criteria, 29 were not available with the literature licenses of Charité and 2 could not be retrieved in English or German. The remaining publications (n=125) were studied with special emphasis on the papers' methods part. 85 papers that gave a good explanation on the isolation were identified, while 22 were excluded because they did not state thoroughly enough, how they obtained hepatocytes and how they purified cell suspensions. Another 18 publications were excluded because the focus on another issue, so that hepatocyte isolation was just a tool to get their experimental materials.

Additionally, 16 papers were included after manual search. These papers were either suggested in references of other publications or recommended at the journals website.

Figure 1 illustrates the literature search strategy and workflow.

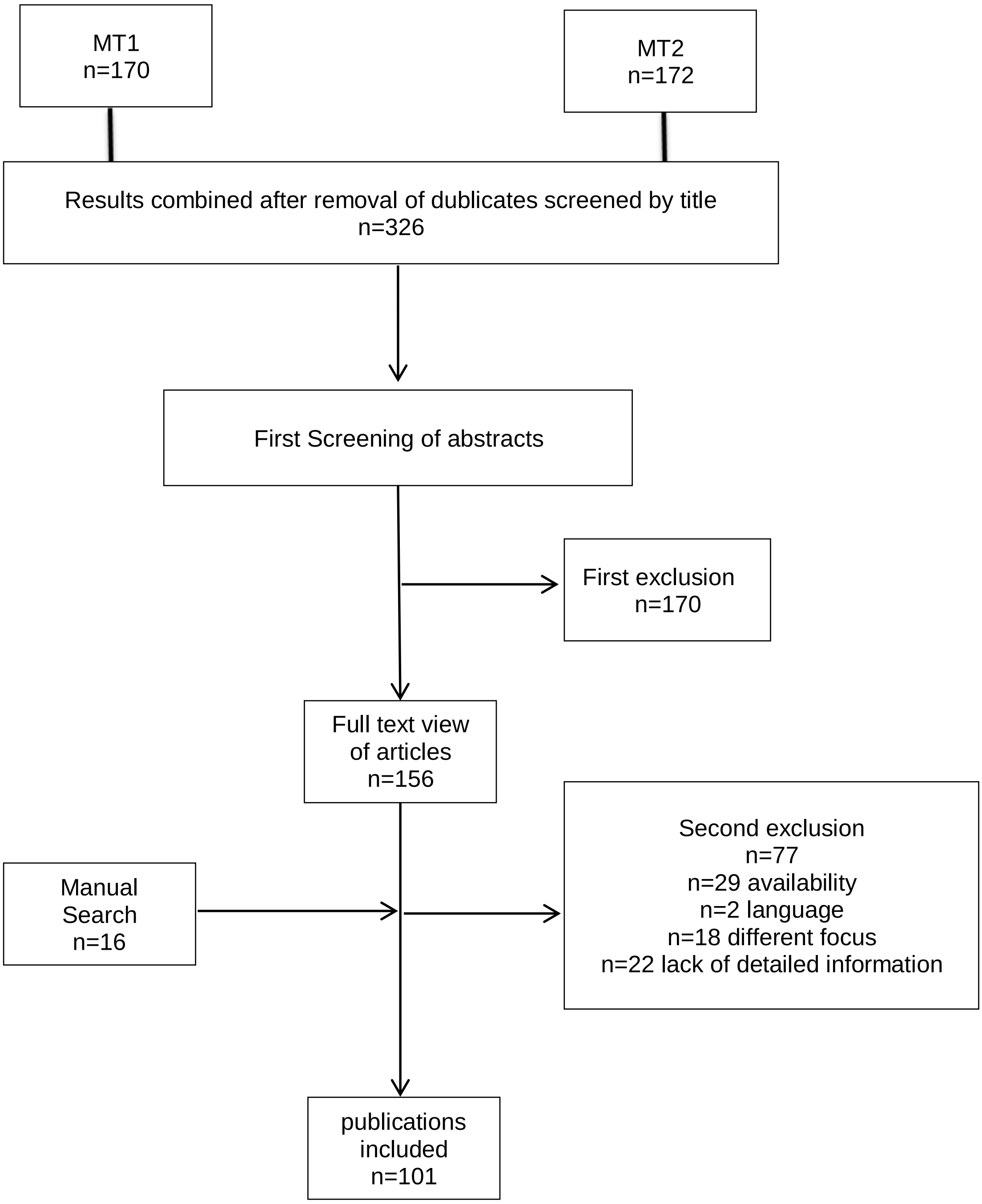

Figure 1: Flow diagram of search strategy

Quality assessment

Resulting papers' quality was assessed using a numeric scoring system. Since an established, published and peer-reviewed quality assessment lacks for evaluation of experimental studies, following questions were considered:

- Is the title adequate?
- Is the abstract adequate and does it accurately reflect the papers objectives?

- Is there an adequate description of statistic methods used and power to meet significance enough?
- Are there any errors in the paper concerning logic or facts?
- Is all the relevant information given to ensure reproducibility?

For each question, 1 point was accounted resulting in a score from 1 (poor quality) to 5 (excellent quality).

Results

101 publications were assessed for this review. Of these, 41 do not use density gradient centrifugation in their protocol, but end with the low speed centrifugation (mostly 50g for 5 minutes).

Figure 4 illustrates the publications reviewed and the different groups with references.

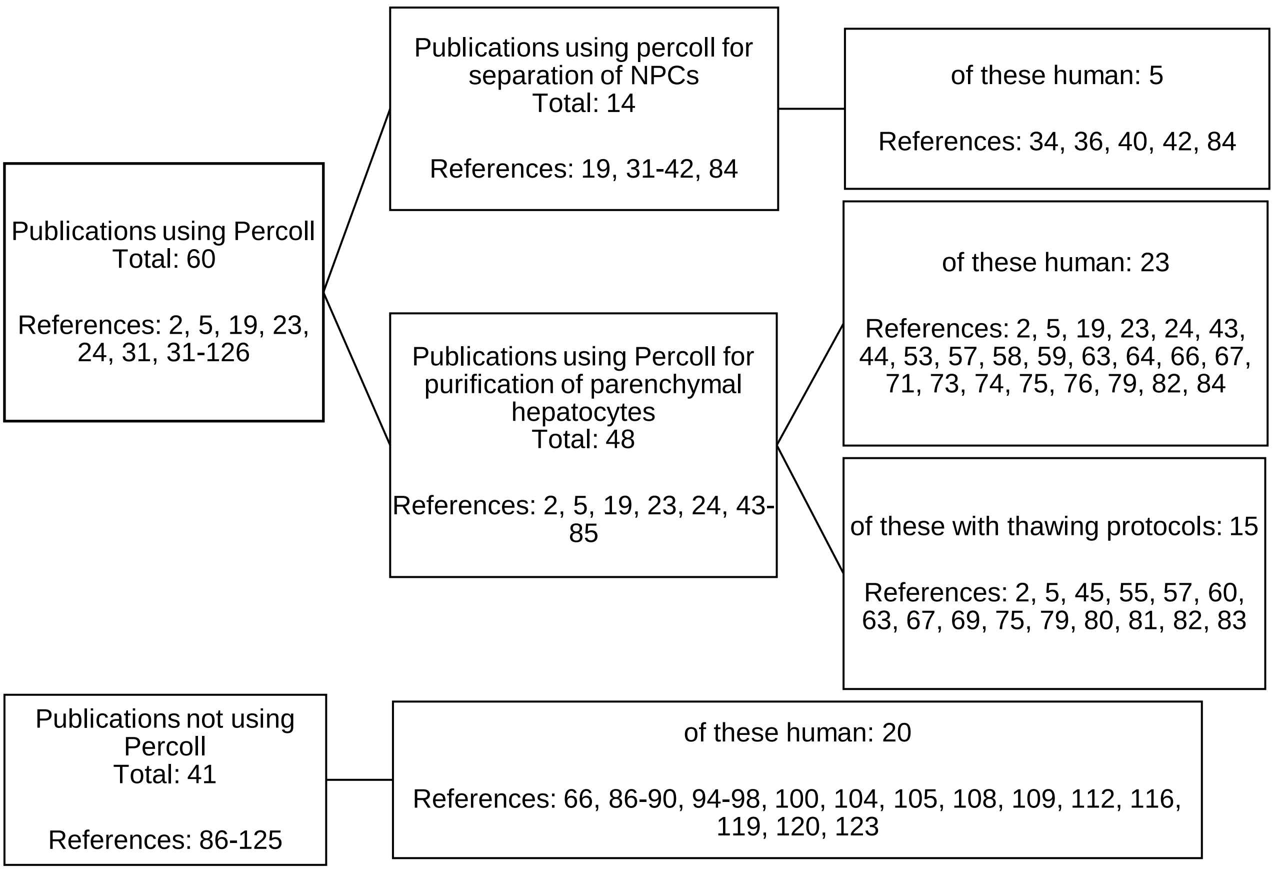

Figure 2. Overview of publications reviewed.

Percoll for purification of Non-Parenchymal cells

Of the 60 publications that mention purification with percoll density gradient in their methods part, 12 use this tool to isolate non-parenchymal cells like Kupffer cells (19, 31, 32, 34, 38, 39, 40), hepatic

progenitor cells (33, 42), oval cells (35) or stellate cells (31, 34, 36, 40); liver sinusoidal endothelial cells (19, 37). These authors use Percoll density gradient layers in different concentrations to separate different cell types of the liver from each other. However, the pellet resulting from the very first centrifugation without density gradient, in which the hepatocytes are concentrated, is not the focus of these works. The supernatant of this step gets centrifuged through several layers of differently concentrated percoll solution. Resulting cultures of NPCs are then cultivated and assessed regarding their function and yield. Detailed information about the pellet with the hepatocytes is missing.

Percoll for purification of parenchymal liver cells

Other 48 publications use percoll density gradient to further purify parenchymal hepatocyte suspensions. Set in relation to the ones not using percoll, 54% of screened publications focusing on hepatocytes use percoll to purify their culture. Of these 48 publications, 15 are using density gradient centrifugation just in conjunction with cryopreservation. Percoll is often used after thawing of freezed primary hepatocytes to split death cells and debris before subsequent use like culturing or transplantation.

Evaluation of purification of hepatocytes with Percoll

The literature search identified only 10 (19, 52, 53, 59, 63, 65, 73, 74, 84, 85) publications which systematically evaluate the benefit of Percoll on isolated hepatocytes, 5 of them (53, 59, 63, 73, 74) with human cells. Table 1 summarizes all information the publications give on their usage of percoll.

According to Schröder et al. in 1994 (74), who performed purification through a 50% concentrated Percoll-suspension for 10min at 50-250g, the reached viability was 94% and the cell culture reached to a high purity. Culture purity was assessed using immunohistological staining and revealed low contamination level of 1%, consisting of fibrocytes. However low yield after percoll purification is reported and in 7 out of 32 experiments, no cells could be recovered after the percoll step.

Olinga et al. (73) published a paper in 2000, in which they investigated, whether parameters of isolated hepatocytes can predict the graft function after transplanting the liver. They compared the yield and viability before and after purification through a Percoll density gradient (they don’t state which concentration). It is reported that yield is significantly decreased (from 13.4 to 4.3 million cells per gram liver tissue) with an average percoll survival rate of 33%, but viability is strongly improved from 68.9 to 92%. They don’t give information on quality and purity of the resulting cells though.

Laba et al. (2005, 59) centrifuged their cell suspensions through a 29-31%-concentrated Percoll layer before and after cryopreservation. Mean viability of crude cells before Percoll purification ranged to 72% and improved to 89% after Percoll. Taken freshly isolated crude cell suspension in comparison with cryopreserved, thawed and then Percoll-purified cells, they found a positive effect

on the plating efficiency after cryopreservation (35-40% improved to 65-85%) and lower levels of non-parenchymal cells when usage of Percoll. Therefore, markers where stained and assessed with immunohistochemistry and FACS: CD3, CD45, CD20, CD90, Ki76 where less positive in percoll-purified cultures, while expression of chemokines was similar in both groups. However, this study is focused on the comparison of fresh and cryopreserved and thawed hepatocytes, there is no metabolic test and limited due to the low number of experiments (n=5).

Yet, these authors do not reflect on the direct comparison on cells before and after Percoll purification regarding cell culture purity and plating efficiency, which are determining parameters for the hepatocytes' subsequent use.

Table 1. Overview of identified publications using percoll to purify parenchymal cells independently of cryopreservation (n=38):

Author	Percoll density gradient usage	species	Reference	Quality score
Bartlett, D. C., et al. (2014). Cytotherapy 16(6): 800-809.	Percoll was used, if initial viability was low and yield sufficiently high. No more detailed information.	human	43	4
Bhogal, R. H. et al. (2011). PloSOne Mar 29;6(3):e18222.	Used if initial viability was <50%. Effect: mean viability improved from 35% to 85%, while yield decreased from 550000 to 175000.	human	44	5
Clouet, A. S., et al. (1998). Analyst 123(12): 2489-2492.	used percoll centrifugation: 8min, 1400g. No more detailed information.	bovine	46	3
Falcieri, E., et al. (1990). Cytotechnology 4(3): 251-259.	After centrifugation through 50% percoll solution, complete purification and removal of debris is reported (less microvilli on cells in REM). No more detailed information.	rats	47	4
Fiegel, H. C., et al. (2000). Tissue Eng 6(6): 619-626.	used percoll 1,13g/ml (95%) 400g, 12min. No more detailed information.	rat	48	4
Foy, B. D., et al. (1994). Biotechnol Bioeng 43(7): 661-672.	used percoll 1.06 g/ml (45%), 50g, 5min, 90% viability. No more detailed information.	rat	49	5
Gerlach, J., et al. (1996). J Surg Res 62(1): 85-89.	used 1.065g/ml (50%) percoll "in selected isolations" viability improved up to 99%. No more detailed information.	pig	50	3
Gerlach, J., et al. (1993). Artif Organs 17(11): 950-953.	percoll improved viability up to a mean of 95%" – No more detailed information.	pig	51	4
Goncalves, L. A., et al. (2007). Malar J 6: 169.	used three different percoll concentrations: 1.12g/ml (99%), 1.08g/ml (60%),1.06g/ml (46%), 750g, 20min. Two sequential density gradient centrifugations. Purity was measured purity with facs: After each gradient centrifugation, there was less contamination with C45RB and CD95.	murine	52	5
Gramignoli, R., et al. (2014). Cell Transplant 23(12): 1545-1556.	Viability improved from 29% to 65% and from 75% to 90% respectively), plating efficiency (10-40% higher), ammonia metabolism and other metabolic activities improved. "Additional studies with cell separation techniques […] seem to be warranted."	human	53	5

Gumucio, J. J., et al. (1986). Hepatology 6(5): 932-944.	used percoll 63%, 40000g, 20min, 4°C; focused on the difference between anterograde and retrograde perfusion techniques.	rat	54	5
Horner, R., et al. (2016). Tissue Eng Part C Methods 22(9): 839-846.	used 25% concentrated percoll solution, 1474g, 20min; no comparison before and after percoll purification.	human	23	5
Johnston, D. E. and R. Jasuja (1994). Hepatology 20(2): 436-444.	Used 54%-percoll layer for purification of parenchymal hepatocytes. NPCs were sedimented between 25% and 50% layer of percoll. No more detailed information about the hepatocyte layer.	rat	56	5
Kawahara, T., et al. (2010). Liver Transpl 16(8): 974-982.	State usage of percoll but no more detailed information.	rat	58	4
Kluge, M., et al. (2016). Tissue Eng Part C Methods 22(1): 38-48.	used 25% concentrated percoll solution, 1474g, 20min; no comparison before and after	human	24	5
Laba, A., et al. (2005). Arch Immunol Ther Exp (Warsz) 53(5): 442-453.	29-31%, Viability improved from 72% to 89%, less contamination with NPCs, better plating efficiency (35-40% to 65-85%)	human	59	4
Odaira, M., et al. (2009). J Surg Res 152(2): 209-217.	Used percoll density gradient to enrich cell viability to at least 90% – no more detailed information.	human	61	5
Ohashi, K., et al. (2012). Cell Transplant 21(2-3): 429-436.	State usage of percoll but no more detailed information.	murine	62	5
Ostrowska, A., et al. (2000). Cell Tissue Bank 1(1): 55-68.	Used percoll at unknown concentration. Puritiy was assessed by CD45 staining, decreased from 8.8% to 0.7% after percoll. Increased viability from 85% to 93%	human	63	5
Puviani, A. C., et al. (1998). Comp Biochem Physiol A Mol Integr Physiol 121(2): 99-109.	State usage of percoll but no more detailed information.	human	64	3
Rajvanshi, P., et al. (1998). Exp Cell Res 244(2): 405-419.	Separated different subpopulations of hepatocytes by their ploidy state through percoll layers of different concentrations: 70%, 52%, 42%, 30%; 1000g, 30min - viablility of parenchymal cells is reported to rise, while yield decreases with increasing percoll concentration. Hepatocytes were split up regarding their state in the azinus before.	rat	65	4

Richert, L., et al. (2004). Liver Int 24(4): 371-378.	Multi-laboratory study. One used percoll, other not. No more detailed information.	human	66	5
Rivas, P. A., et al. (1993). Transplantation 55(2): 335-339.	State usage of percoll but no more detailed information.	rabbit	68	4
Shulman, M. and Y. Nahmias (2013). Methods Mol Biol 945: 287-302.	500g, 5min; but no more detailed information.	rat	70	5
Vondran, F. W., et al. (2008). Artif Organs 32(3): 205-213.	Use percoll: 25%, 1278g, 20min. Viability was risen from 70.3 to 82.5%, while yield was decreased from 18.7 to 13.2x10^6/g, percoll survival 68%	human	71	5
Wang, H., et al. (1998). J Biochem 124(5): 892-899.	Use percoll 45%, microscopically assessed purity was 99% and viablility was 98% measured by Trypan blue exclusion. No comparison before and after percoll.	murine	72	4
Smedsrød B, Pertoft H. (1985). J Leukoc Biol. 38(2):213-30	Used percoll for primary hepatocytes: 1.08% (60%), 50g, 2min; supernatant in different percoll-concentrations for NPCs. After 60% density centrifugation, PCs are stated to be nearly 100% viable and no detectable contaminating NPCs. 50-60% percoll survival.	rat	19	5
Olinga P, et al. (2000). Liver. 20(5):374-80.	Percoll concentration not stated. Focus more on the correlation of hepatocyte isolation with clinical features of the donor. Mean Percoll survival: 33%; mean yield was decreased from 13.4 to 4.3million cells/g tissue while viability was increased from 68.9 to 92% on average.	human	73	5
Schröder AJ, et al (1994). Zentralbl Chir., 119(2):127-38	50% percoll, 10min, 50-250g; Contamination level of NPCs =1% (fibrocytes); report low yield after percoll-purification (in 7 out of 32 experiments no outcome after percoll). Assessment of purity with immunohistochemical staining. No metabolical assay.	human	74	5
Godoy P et al. (2013). Arch Toxicol. 87(8):1315-530	Recommend 25%-concentrated percoll, 1200-1200g for 15-20min for the best separation between viable and dead cells. Recommend 48%-concentrated percoll, 28g for 5min to purify hepatocytes. No comparison.	Human/ rat	5	4
Li AP. (2007). Chem Biol Interact 168(1):16-29	State usage of percoll but no more detailed information about it.	human	75	4

Knobeloch D, et al. (2012). Methods Mol Biol. 806:99-120	Use percoll 25%, 1278g, 20min; no more detailed information about it.	human	76	4
Ehrhard S, et al. (2016). EXCLI J. 15:858-866.	Use percoll 1.124g/ml (92%), 1000g, no more detailed information.	bovine	77	4
Spotorno VG, et al. (2006). Cytotechnology. 51(2):51-6	Non-perfusion-technique. Compare before and after 60% percoll - metabolic activity higher – When Percoll purification was performed, only larger cells were selected with a lower yield. These cells were not as confluent but metabolically more active.	bovine	78	5
McGinnity DF, et al. (2004). Drug Metab Dispos. 32(11):1247-53.	25% Percoll purification increased dog hepatocyte viability from 52 to 78% and human hepatocyte viability from 66 to 88%. “Percoll concentrations above 25% led to an unacceptable loss of cells in both species.”	Human/ dog	79	5
Innes GK, et al. (1988). Cryobiology 25(1):23-30	Percoll density 1.07g/ml (55%) and 1.02g/ml (12.5%), 30000g, 90min, was performed and two separate populations of hepatocytes were obtained this way. “One denser than the other with significantly greater trypan blue dye-exclusion capabilities (P < 0.05).” Purified cells through the higher-concentrated Percoll layer performed better metabolically and had a better membrane integrity than the less dense cells.	rat	80	5
Tateno C, et al. (2000). Hepatology 31(1):65-74	Used Percoll concentrated 45%, 50g, 10min; separated different subpopulations of hepatocytes (“normal” vs “small hepatocytes”) and NPCs. Assessed effects of different co-cultures and growth potential and purity with FACS and immunohistochemistry. Focus is not on one population of parenchymal cells before and after density centrifugation.	rat	84	5
Kreamer BL, et al. (1986). 22(4):201-11.	Used two different density gradient protocols, one iso-density with 1.06g/ml (45%), 50g, 10min. Viability was improved from 87.1 to 98%. Rat hepatocytes purified this way performed better metabolically (cytochrome p-450, tyrosine aminotransferase, glutamic-oxaloacetic transaminase, hormone response). Percoll survival was 90%, cells sowed better plating efficiency, and a higher percentage of single cells, less debris and almost no NPCs. Also subsequent viability is reported to be better	rat	85	4

References

1. Russell WMS, Burch RL, The principles of humane experimental technique. Methuen and Co ltd., United Kingdom, 1959

2. Hengstler, J.G., Utesch D, Steinberg P et al., Cryopreserved primary hepatocytes as a constantly available in vitro model for the evaluation of human and animal drug metabolism and enzyme induction. Drug metabolism reviews 2000;32(1):81-118

3. Gómez-Lechón MJ, Castell JV, Donato MT. The use of hepatocytes to investigate drug toxicity. Methods Mol Biol 2010;640:389-415

4. Li A.P, Jurima-Romet M. Applications of primary hepatocytes in the evaluation of pharmacokinetic drug-drug-interactions: evaluation of model drugs terfenadine and rifampin. Cell Biol Toxicol 1997;13:365

5. Godoy P, Hewitt NJ, Albrecht U et al. Recent advances in 2D and 3D in vitro systems using primary hepatocytes, alternative hepatocyte sources and non-parenchymal liver cells and their use in investigating mechanisms of hepatotoxicity, cell signaling and ADME. Arch Toxicol. 2013 Aug;87(8):1315-530

6. Ratziu V, Belletani S, Cortez-Pinto H, Day C Marchesini G. A position statement on NAFLD/NASH based on the EASL 2009 special conference. J Hepatol. 2010;53(2):372-84

7. Wong RJ, Aguilar M, Cheung R, et al. Nonalcoholic steatohepatitis is the second leading etiology of liver disease among adults awaiting liver transplantation in the United States. Gastroenterology. 2015 Mar;148(3):547-55

8. Pais R, Barritt AST, Calmus Y, et al. NAFLD and liver transplantation: Current burden and expected challenges. J Hepatol. 2016;65(6):1245-57

9. Strom SC, Fisher RA, Thompson MT, Sanyal AJ, Cole PE, Ham JM, Posner MP. Hepatocyte transplantation as a bridge to orthotopic liver transplantation in terminal liver failure. Transplantation. 1997;63(4):559-69

10. Struecker B, Raschzok N, Sauer IM. Liver support strategies: cutting-edge technologies. Nat Rev Gastroenterol Hepatol. 2014;11(3):166-76

11. Fox IJ, Chowdhury JR, Kaufman SS, et al. Treatment of the Crigler-Najjar syndrome type I with hepatocyte transplantation. N. Engl. J. Med. 1998;338:1422–1426.

12. Hughes RD, Mitry RR, Dhawan A. Current status of hepatocyte transplantation. Transplantation 2012;93:342–347

13. Mazza G, Rombouts K, Rennie Hall A, et al. Decellularized human liver as a natural 3D-scaffold for liver bioengineering and transplantation. Sci Rep. 2015;5:13079.

14. Uygun BE, Yarmush ML, Uygun K. Application of whole-organ tissue engineering in hepatology. Nature reviews Gastroenterology & hepatology 2012;9(12):738-744.

15. Shirakigawa N, Ijima H, Takei T. Decellularized liver as a practical scaffold with a vascular network template for liver tissue engineering. J Biosci Bioeng. 2012 Nov;114(5):546-51

16. Howard RB, Christensen AK, Gibbs FA, Pesch LA. The enzymatic preparation of isolated intact parenchymal cells from rat liver.J Cell Biol. 1967 Dec;35(3):675-84.

17. Seglen PO. Preparation of isolated rat liver cells. Methods Cell Biol. 1976;13:29-83.

18. Strom SC, Jirtle RL, Jones RS et al. Isolation, culture, and transplantation of human hepatocytes. J Natl Cancer Inst. 1982;68(5):771-8

19. Smedsrød B, Pertoft H. Preparation of pure hepatocytes and reticuloendothelial cells in high yield from a single rat liver by means of Percoll centrifugation and selective adherence. J Leukoc Biol. 1985 Aug;38(2):213-30.

20. Hewitt WR, Corno V, Eguchi S, et al. Isolation of human hepatocytes from livers rejected for whole organ transplantation. Transplant Proc 1997;29(4):1945-7

21. Baccarani U, Donini A, Risaliti A, et al. Steatotic versus cirrhotic livers as a source for human hepatocyte isolation. Transplant Proc 2001;33(1-2):664-5

22. Pertoft H, Laurent TC, Låås T, Kågedal L. Density gradients prepared from colloidal silica particles coated by polyvinylpyrrolidone (Percoll). Anal Biochem. 1978;88(1):271-82

23. Horner R, Kluge M, Gassner J, et al. Hepatocyte Isolation After Laparoscopic Liver Resection Tissue Eng Part C Methods. 2016 Sep;22(9):839-46.

24. Kluge M, Reutzel-Selke A, Napierala H, et al. Human Hepatocyte Isolation: Does Portal Vein Embolization Affect the Outcome?. Tissue Eng Part C Methods. 2016;22(1):38-48

25. Hansel MC, Davila J, Vosough M, et al. The Use of Induced Pluripotent Stem Cells for the Study and Treatment of Liver Diseases. Curr Protoc Toxicol. 2016 Feb 1;67:14.13.1-14.13.27.

26. Khan, A.A., Shaik, M.V., Parveen, N., et al. Human fetal liver-derived stem cell transplantation as supportive modality in the management of end-stage decompensated liver cirrhosis. Cell Transplant 2010;19:409

27. Cardinale V, Carpino G., Gentile, R., et al. Transplantation of human fetal biliary tree stem/progenitor cells into two patients with advanced liver cirrhosis. BMC Gastroenterol 2014;14:204

28. Tsolaki E, Yannaki E. Stem cell-based regenerative opportunities for the liver: State of the art and beyond. World Journal of Gastroenterology 2015;21(43):12334-12350.

29. Vinken M, Vanhaecke T, Rogiers V. Primary hepatocyte cultures as in vitro tools for toxicity testing: quo vadis?. Toxicol In Vitro. 2012;26(3):541-4.

30. Clavien PA. Sinusoidal endothelial cell injury during hepatic preservation and reperfusion. Hepatology 1998;28:281-5

31. Kegel V, Deharde D, Pfeiffer E, Zeilinger K, Seehofer D, Damm G. Protocol for Isolation of Primary Human Hepatocytes and Corresponding Major Populations of Non-parenchymal Liver Cells. Journal of visualized experiments: JoVE. 2016(109):e53069.

32. Chen X, Xu C. Transcription Profiles of Marker Genes Predict The Transdifferentiation Relationship between Eight Types of Liver Cell during Rat Liver Regeneration. Cell journal. 2015;17(2):339-54.

33. Bourgognon M, Klippstein R, Al-Jamal KT. Kupffer Cell Isolation for Nanoparticle Toxicity Testing. Journal of visualized experiments: JoVE. 2015(102):e52989.

34. Guo J, Li J, Lu Y, Xin J, Jiang L, Wu W, et al. A novel technique for hepatic progenitor cell isolation from normal adult rat livers. ASAIO journal (American Society for Artificial Internal Organs: 1992;2012;58(1):73-8.

35. Koenig S, Probst I, Becker H, Krause P. Zonal hierarchy of differentiation markers and nestin expression during oval cell mediated rat liver regeneration. Histochemistry and cell biology. 2006;126(6):723-34.

36. Lee SM, Schiergens TS, Demmel M, Thasler RM, Thasler WE. Isolation of Hepatocytes and Stellate Cells from a Single Piece of Human Liver. Methods in molecular biology (Clifton, NJ). 2017;1506:247-58.

37. Meyer J, Lacotte S, Morel P, Gonelle-Gispert C, Buhler L. An optimized method for mouse liver sinusoidal endothelial cell isolation. Experimental cell research. 2016;349(2):291-301.

38. Mohar I, Brempelis KJ, Murray SA, Ebrahimkhani MR, Crispe IN. Isolation of Non-parenchymal Cells from the Mouse Liver. Methods in molecular biology (Clifton, NJ). 2015;1325:3-17.

39. Olynyk JK, Clarke SL. Isolation and primary culture of rat Kupffer cells. Journal of gastroenterology and hepatology. 1998;13(8):842-5.

40. Pfeiffer E, Kegel V, Zeilinger K, Hengstler JG, Nussler AK, Seehofer D, et al. Featured Article: Isolation, characterization, and cultivation of human hepatocytes and non-parenchymal liver cells. Experimental biology and medicine (Maywood, NJ). 2015;240(5):645-56.

41. Riccalton-Banks L, Bhandari R, Fry J, Shakesheff KM. A simple method for the simultaneous isolation of stellate cells and hepatocytes from rat liver tissue. Molecular and cellular biochemistry. 2003;248(1-2):97-102.

42. Tolosa L, Bonora-Centelles A, Teresa Donato M, Pareja E, Negro A, Lopez S, et al. Steatotic liver: a suitable source for the isolation of hepatic progenitor cells. Liver international : official journal of the International Association for the Study of the Liver. 2011;31(8):1231-8.

43. Bartlett DC, Hodson J, Bhogal RH, Youster J, Newsome PN. Combined use of N-acetylcysteine and Liberase improves the viability and metabolic function of human hepatocytes isolated from human liver. Cytotherapy. 2014;16(6):800-9.

44. Bhogal RH, Hodson J, Bartlett DC, et al. Isolation of primary hepatocytes from normal and diseased liver tissue: a one hundred liver experience. PLoS One. 2011 Mar 29;6(3):e18222.

45. Chesne C, Guillouzo A. Cryopreservation of isolated rat hepatocytes: a critical evaluation of freezing and thawing conditions. Cryobiology. 1988;25(4):323-30.

46. Clouet AS, Le Bizec B, Boerlen F, Monteau F, Andre F. Calf primary hepatocyte culture as a tool for anabolic steroid metabolism studies. The Analyst. 1998;123(12):2489-92.

47. Falcieri E, Del Coco R, Mariani AR, Gobbi P, Santi P. Membrane modifications in the course of hepatocyte isolation. Cytotechnology. 1990;4(3):251-9.

48. Fiegel HC, Kaufmann PM, Kneser U, Kluth D, Rogiers X. Priming of hepatocytes for cell culture by partial hepatectomy prior to cell isolation. Tissue engineering. 2000;6(6):619-26.

49. Foy BD, Toner M, Tompkins RG, Yarmush ML. Engineering organ perfusion protocols: NMR analysis of hepatocyte isolation from perfused rat liver. Biotechnology and bioengineering. 1994;43(7):661-72.

50. Gerlach J, Brombacher J, Smith M, Neuhaus P. High yield hepatocyte isolation from pig livers for investigation of hybrid liver support systems: influence of collagenase concentration and body weight. The Journal of surgical research. 1996;62(1):85-9.

51. Gerlach J, Kloppel K, Schon MR, Brombacher J, Courtney JM, Unger J, et al. Comparison of pig hepatocyte isolation using intraoperative perfusion without warm ischemia and isolation of cells from abattoir organs after warm ischemia. Artificial organs. 1993;17(11):950-3.

52. Goncalves LA, Vigario AM, Penha-Goncalves C. Improved isolation of murine hepatocytes for in vitro malaria liver stage studies. Malaria journal. 2007;6:169.

53. Gramignoli R, Tahan V, Dorko K, Venkataramanan R, Fox IJ, Ellis EC, et al. Rapid and sensitive assessment of human hepatocyte functions. Cell transplantation. 2014;23(12):1545-56.

54. Gumucio JJ, May M, Dvorak C, Chianale J, Massey V. The isolation of functionally heterogeneous hepatocytes of the proximal and distal half of the liver acinus in the rat. Hepatology (Baltimore, Md). 1986;6(5):932-44.

55. Izamis ML, Perk S, Calhoun C, Uygun K, Yarmush ML, Berthiaume F. Machine perfusion enhances hepatocyte isolation yields from ischemic livers. Cryobiology. 2015;71(2):244-55.

56. Johnston DE, Jasuja R. Purification of cultured primary rat hepatocytes using selection with ricin A subunit. Hepatology (Baltimore, Md). 1994;20(2):436-44.

57. Katenz E, Vondran FW, Schwartlander R, Pless G, Gong X, Cheng X, et al. Cryopreservation of primary human hepatocytes: the benefit of trehalose as an additional cryoprotective agent. Liver transplantation : official publication of the American Association for the Study of Liver Diseases and the International Liver Transplantation Society. 2007;13(1):38-45.

58. Kawahara T, Toso C, Douglas DN, Nourbakhsh M, Lewis JT, Tyrrell DL, et al. Factors affecting hepatocyte isolation, engraftment, and replication in an in vivo model. Liver transplantation : official publication of the American Association for the Study of Liver Diseases and the International Liver Transplantation Society. 2010;16(8):974-82.

59. Laba A, Ostrowska A, Patrzalek D, Paradowski L, Lange A. Characterization of human hepatocytes isolated from non-transplantable livers. Archivum immunologiae et therapiae experimentalis. 2005;53(5):442-53.

60. Mei J, Sgroi A, Mai G, Baertschiger R, Gonelle-Gispert C, Serre-Beinier V, et al. Improved survival of fulminant liver failure by transplantation of microencapsulated cryopreserved porcine hepatocytes in mice. Cell transplantation. 2009;18(1):101-10.

61. Odaira M, Aoki T, Miyamoto Y, Yasuhara R, Jin Z, Yu J, et al. Cold preservation of the liver with oxygenation by a two-layer method. The Journal of surgical research. 2009;152(2):209-17.

62. Ohashi K, Tatsumi K, Tateno C, Kataoka M, Utoh R, Yoshizato K, et al. Liver tissue engineering utilizing hepatocytes propagated in mouse livers in vivo. Cell transplantation. 2012;21(2-3):429-36.

63. Ostrowska A, Bode DC, Pruss J, Bilir B, Smith GD, Zeisloft S. Investigation of functional and morphological integrity of freshly isolated and cryopreserved human hepatocytes. Cell and tissue banking. 2000;1(1):55-68.

64. Puviani AC, Ottolenghi C, Tassinari B, Pazzi P, Morsiani E. An update on high-yield hepatocyte isolation methods and on the potential clinical use of isolated liver cells. Comparative biochemistry and physiology Part A, Molecular & integrative physiology. 1998;121(2):99-109.

65. Rajvanshi P, Liu D, Ott M, Gagandeep S, Schilsky ML, Gupta S. Fractionation of rat hepatocyte subpopulations with varying metabolic potential, proliferative capacity, and retroviral gene transfer efficiency. Experimental cell research. 1998;244(2):405-19.

66. Richert L, Alexandre E, Lloyd T, Orr S, Viollon-Abadie C, Patel R, et al. Tissue collection, transport and isolation procedures required to optimize human hepatocyte isolation from waste liver surgical resections. A multilaboratory study. Liver international: official journal of the International Association for the Study of the Liver. 2004;24(4):371-8.

67. Richert L, Liguori MJ, Abadie C, Heyd B, Mantion G, Halkic N, et al. Gene expression in human hepatocytes in suspension after isolation is similar to the liver of origin, is not affected by hepatocyte cold storage and cryopreservation, but is strongly changed after hepatocyte plating. Drug metabolism and disposition: the biological fate of chemicals. 2006;34(5):870-9.

68. Rivas PA, Fabrega AJ, Schwartz D, Pollak R. The morphology and function of rabbit hepatocytes isolated using ethylenediaminetetraacetate. Transplantation. 1993;55(2):335-9.

69. Shibany KA, Totemeyer S, Pratt SL, Paine SW. Equine hepatocytes: isolation, cryopreservation, and applications to in vitro drug metabolism studies. Pharmacology research & perspectives. 2016;4(5):e00268.

70. Shulman M, Nahmias Y. Long-term culture and coculture of primary rat and human hepatocytes. Methods in molecular biology (Clifton, NJ). 2013;945:287-302.

71. Vondran FW, Katenz E, Schwartlander R, Morgul MH, Raschzok N, Gong X, et al. Isolation of primary human hepatocytes after partial hepatectomy: criteria for

identification of the most promising liver specimen. Artificial organs. 2008;32(3):205-13.

72. Wang H, Gao X, Fukumoto S, Tademoto S, Sato K, Hirai K. Post-isolation inducible nitric oxide synthase gene expression due to collagenase buffer perfusion and characterization of the gene regulation in primary cultured murine hepatocytes. Journal of biochemistry. 1998;124(5):892-9.

73. Olinga P, Maring JK, Merema M, et al., The capability of isolated hepatocytes and liver slices of donor livers to predict graft function after liver transplantation. Liver. 2000 Oct;20(5):374-80.

74. Schröder AJ, Blaheta RA, Scholz M, Encke A, Markus BH. Isolation and separation of human adult hepatocytes from resected liver: cellular yield and purity using different methods. Zentralbl Chir., 1994, 119(2):127-38

75. Li AP. Human hepatocytes: Isolation, cryopreservation and applications in drug development. Chem Biol Interact. 2007 May 20;168(1):16-29

76. Knobeloch D, Ehnert S, Schyschka L, et al. Human Hepatocytes: Isolation, Culture, and Quality Procedures. Methods Mol Biol. 2012;806:99-120

77. Ehrhard S, Schmicke M. Isolation and cultivation of adult primary bovine hepatocytes from abattoir derived liver. EXCLI J. 2016 Dec 22;15:858-866.

78. Spotorno VG, Hidalgo A, Barbich M, Lorenti A, Zabal O. Culture of bovine hepatocytes: a non-perfusion technique for cell isolation. Cytotechnology. 2006 Jun;51(2):51-6.

79. McGinnity DF, Soars MG, Urbanowicz RA, Riley RJ. Evaluation of fresh and cryopreserved hepatocytes as in vitro drug metabolism tools for the prediction of metabolic clearance. Drug Metab Dispos. 2004 Nov;32(11):1247-53.

80. Innes GK, Fuller BJ, Hobbs KE. Functional testing of hepatocytes following their recovery from cryopreservation. Cryobiology. 1988 Feb;25(1):23-30

81. Diener B, Utesch D, Beer N, Dürk H, Oesch F. A method for the cryopreservation of liver parenchymal cells for studies of xenobiotics. Cryobiology. 1993 Apr;30(2):116-27.

82. Dou M, de Sousa G, Lacarelle B, et al. Thawed human hepatocytes in primary culture. Cryobiology. 1992 Aug;29(4):454-69

83. Utesch D, Diener B, Molitor E, Oesch F, Platt KL. Characterization of cryopreserved rat liver parenchymal cells by metabolism of diagnostic substrates and activities of related enzymes. Biochem Pharmacol. 1992 Jul 22;44(2):309-15.

84. Tateno C, Takai-Kajihara K, Yamasaki C, Sato H, Yoshizato K. Heterogeneity of growth potential of adult rat hepatocytes in vitro. Hepatology. 2000 Jan;31(1):65-74

85. Kreamer BL, Staecker JL, Sawada N, Sattler GL, Hsia MT, Pitot HC. Use of a low-speed, iso-density percoll centrifugation method to increase the viability of isolated rat hepatocyte preparations. In Vitro Cell Dev Biol. 1986 Apr;22(4):201-11.

86. Alexandre E, Cahn M, Abadie-Viollon C, Meyer N, Heyd B, Mantion G, et al. Influence of pre-, intra- and post-operative parameters of donor liver on the outcome of isolated human hepatocytes. Cell and tissue banking. 2002;3(4):223-33.

87. Alexandre E, Viollon-Abadie C, David P, Gandillet A, Coassolo P, Heyd B, et al. Cryopreservation of adult human hepatocytes obtained from resected liver biopsies. Cryobiology. 2002;44(2):103-13.

88. Baccarani U, Donini A, Risaliti A, Piccolo G, Dialti V, Cautero N, et al. Steatotic versus cirrhotic livers as a source for human hepatocyte isolation. Transplantation proceedings. 2001;33(1-2):664-5.

89. Baccarani U, Sainz-Barriga M, Adani GL, Risaliti A, Bresadola F, Donini A, et al. Human hepatocyte isolation for liver cell therapy: whole marginal livers or healthy segments from splitting? Transplantation. 2005;79(2):249; author reply 50.

90. Baccarani U, Sanna A, Cariani A, Sainz-Barriga M, Adani GL, Zambito AM, et al. Isolation of human hepatocytes from livers rejected for liver transplantation on a national basis: results of a 2-year experience. Liver transplantation: official publication of the American Association for the Study of Liver Diseases and the International Liver Transplantation Society. 2003;9(5):506-12.

91. Bakala A, Karlik W, Wiechetek M. Preparation of equine isolated hepatocytes. Toxicology in vitro : an international journal published in association with BIBRA. 2003;17(5-6):615-21.

92. Donini A, Baccarani U, Piccolo G, Lavaroni S, Dialti V, Cautero N, et al. Hepatocyte isolation using human livers discarded from transplantation: analysis of cell yield and function. Transplantation proceedings. 2001;33(1-2):654-5.

93. Fautz R, Husein B, Efstathiou E, Hechenberger-Freudl C. Assessment of the relation between the initial viability and the attachment of freshly isolated rat hepatocytes used for the in vivo/in vitro DNA repair assay (UDS). Mutation research. 1993;291(1):21-7.

94. Fontaine M, Schloo B, Jenkins R, Uyama S, Hansen L, Vacanti JP. Human hepatocyte isolation and transplantation into an athymic rat, using prevascularized cell polymer constructs. Journal of pediatric surgery. 1995;30(1):56-60.

95. Foquet L, Hermsen CC, van Gemert GJ, Libbrecht L, Sauerwein R, Meuleman P, et al. Molecular detection and quantification of Plasmodium falciparum-infected human hepatocytes in chimeric immune-deficient mice. Malaria journal. 2013;12:430.

96. Gramignoli R, Green ML, Tahan V, Dorko K, Skvorak KJ, Marongiu F, et al. Development and application of purified tissue dissociation enzyme mixtures for human hepatocyte isolation. Cell transplantation. 2012;21(6):1245-60.

97. Hang H, Shi X, Gu G, Wu Y, Ding Y. A simple isolation and cryopreservation method for adult human hepatocytes. The International journal of artificial organs. 2009;32(10):720-7.

98. Hossein Aghdaie M, Geramizadeh B, Azarpira N, Esfandiari E, Darai M, Rahsaz M, et al. Hepatocyte isolation from unused/rejected livers for transplantation: initial step toward hepatocyte transplantation, the first experience from iran. Hepatitis monthly. 2013;13(8):e10397.

99. Hsu HC, Matsuno N, Machida N, Enosawa S. Improved Recovery of Hepatocytes Isolated From Warm Ischemic Rat Liver by Citrate Phosphate Dextrose (CPD)-Supplemented Euro-Collins Solution. Cell medicine. 2013;5(2-3):97-101.

100. Ibars EP, Cortes M, Tolosa L, Gomez-Lechon MJ, Lopez S, Castell JV, et al. Hepatocyte transplantation program: Lessons learned and future strategies. World journal of gastroenterology. 2016;22(2):874-86.

101. Klaunig JE, Goldblatt PJ, Hinton DE, Lipsky MM, Chacko J, Trump BF. Mouse liver cell culture. I. Hepatocyte isolation. In vitro. 1981;17(10):913-25.

102. Lanford RE, Carey KD, Estlack LE, Smith GC, Hay RV. Analysis of plasma protein and lipoprotein synthesis in long-term primary cultures of baboon hepatocytes maintained in serum-free medium. In vitro cellular & developmental biology: journal of the Tissue Culture Association. 1989;25(2):174-82.

103. Lawrence JN, Foster B, Benford DJ. The application of a wedge perfusion technique to the in vivo-in vitro rat hepatocyte DNA-repair assay. Mutation research. 1991;252(2):129-37.

104. Lehec SC. Microbiological monitoring of hepatocyte isolation in the GMP laboratory. Methods in molecular biology (Clifton, NJ). 2009;481:221-7.

105. Lehec SC, Hughes RD, Mitry RR, Graver MA, Verma A, Wade JJ, et al. Experience of microbiological screening of human hepatocytes for clinical transplantation. Cell transplantation. 2009;18(8):941-7.

106. Li Y, Wang Y, Wu Q, Li L, Shi Y, Bu H, et al. Comparison of methods for isolating primary hepatocytes from mini pigs. Xenotransplantation. 2016;23(5):414-20.

107. Meng FY, Chen ZS, Han M, Hu XP, He XX, Liu Y, et al. Porcine hepatocyte isolation and reversible immortalization mediated by retroviral transfer and site-specific recombination. World journal of gastroenterology. 2010;16(13):1660-4.

108. Meng FY, Liu L, Yang FH, Li CY, Liu J, Zhou P. Reversible immortalization of human hepatocytes mediated by retroviral transfer and site-specific recombination. World journal of gastroenterology. 2014;20(36):13119-26.

109. Mitry RR, Hughes RD, Aw MM, Terry C, Mieli-Vergani G, Girlanda R, et al. Human hepatocyte isolation and relationship of cell viability to early graft function. Cell transplantation. 2003;12(1):69-74.

110. Moir RD, Smith GJ, Stewart BW. Structural defects in DNA from primary hepatocyte cultures. Cell biology international reports. 1983;7(3):227-36.

111. Nelson LJ, Newsome PN, Howie AF, Hadoke PW, Dabos KJ, Walker SW, et al. An improved ex vivo method of primary porcine hepatocyte isolation for use in bioartificial liver systems. European journal of gastroenterology & hepatology. 2000;12(8):923-30.

112. Noormohamed MS, Kanwar A, Ray C, Wright MC, Cowie DE, Stamp S, et al. Extracorporeal membrane oxygenation for resuscitation of deceased cardiac donor livers for hepatocyte isolation. The Journal of surgical research. 2013;183(2):e39-48.

113. Okubo H, Matsushita M, Kamachi H, Kawai T, Takahashi M, Fujimoto T, et al. A novel method for faster formation of rat liver cell spheroids. Artificial organs. 2002;26(6):497-505.

114. Pinkse GG, Voorhoeve MP, Noteborn M, Terpstra OT, Bruijn JA, De Heer E. Hepatocyte survival depends on beta1-integrin-mediated attachment of hepatocytes to

hepatic extracellular matrix. Liver international : official journal of the International Association for the Study of the Liver. 2004;24(3):218-26.

115. Porretti L, Gatti S, Gramignoli R, Colombo F, Lopa R, Cattaneo A, et al. Animal model for liver cell banking from non-heart beating donors after prolonged ischaemia time. Digestive and liver disease : official journal of the Italian Society of Gastroenterology and the Italian Association for the Study of the Liver. 2006;38(12):905-11.

116. Riou JP, Audigier C, Laville M, Beylot M, Pigeon P, Mornex R. Dephosphorylation of L-pyruvate kinase during rat liver hepatocyte isolation. Archives of biochemistry and biophysics. 1985;236(1):321-7.

117. Rodriguez-Ariza A, Paine AJ. Rapid induction of NF-kappaB binding during liver cell isolation and culture: inhibition by L-NAME indicates a role for nitric oxide synthase. Biochemical and biophysical research communications. 1999;257(1):145-8.

118. Sagias FG, Mitry RR, Hughes RD, Lehec SC, Patel AG, Rela M, et al. N-acetylcysteine improves the viability of human hepatocytes isolated from severely steatotic donor liver tissue. Cell transplantation. 2010;19(11):1487-92.

119. Serralta A, Donato MT, Orbis F, Castell JV, Mir J, Gomez-Lechon MJ. Functionality of cultured human hepatocytes from elective samples, cadaveric grafts and hepatectomies. Toxicology in vitro: an international journal published in association with BIBRA. 2003;17(5-6):769-74.

120. Severgnini M, Sherman J, Sehgal A, Jayaprakash NK, Aubin J, Wang G, et al. A rapid two-step method for isolation of functional primary mouse hepatocytes: cell characterization and asialoglycoprotein receptor based assay development. Cytotechnology. 2012;64(2):187-95.

121. Smets FN, Chen Y, Wang LJ, Soriano HE. Loss of cell anchorage triggers apoptosis (anoikis) in primary mouse hepatocytes. Molecular genetics and metabolism. 2002;75(4):344-52.

122. Solanas E, Sostres C, Serrablo A, Garcia-Gil A, Garcia JJ, Aranguren FJ, et al. Effect of Dimethyl Sulfoxide and Melatonin on the Isolation of Human Primary Hepatocytes. Cells, tissues, organs. 2015;200(5):316-25.

123. Tormos AM, Talens-Visconti R, Bonora-Centelles A, Perez S, Sastre J. Oxidative stress triggers cytokinesis failure in hepatocytes upon isolation. Free radical research. 2015;49(8):927-34.

124. Tormos Ana M, Talens-Visconti R, Jorques M, Perez-Garrido S, Bonora-Centelles A, Nebreda Angel R, et al. p38alpha deficiency and oxidative stress cause cytokinesis failure in hepatocytes. Free radical biology & medicine. 2014;75 Suppl 1:S19.

125. van de Kerkhove MP, Poyck PP, van Wijk AC, Galavotti D, Hoekstra R, van Gulik TM, et al. Assessment and improvement of liver specific function of the AMC-bioartificial liver. The International journal of artificial organs. 2005;28(6):617-30.

126. Lee SM., et al., An algorithm that predicts the Viability and yield of Human Hepatocytes Isolated from Remnant Liver Pieces Obtained from Liver Resections, 2014, PLoS one

127. Hewitt WR, Corno V, Eguchi S., et al., Isolation of human hepatocytes from livers rejected for whole organ transplantation. Transplant Proc 1997;29(4):1945-7

128. Sauer IM, Obermeyer N, Kardassis D, Theruvat T, Gerlach JC. Development of a hybrid liver support system. Ann. NY Acad. Sci. 2001;944,308-319

129. Uygun BE, Yarmush ML. Engineered liver for transplantation. Curr Opin Biotechnol. 2013 Oct;24(5):893-9.

130. Uygun BE, Soto-Gutierrez A, Yagi H, et al. Organ reengineering through development of a transplantable recellularized liver graft using decellularized liver matrix. Nat Med. 2010 Jul;16(7):814-20.

131. Zhou P, Lessa N, Estrada DC, Severson EB, Lingala S, Zern MA, Nolta JA, Wu J. Decellularized liver matrix as a carrier for the transplantation of human fetal and primary hepatocytes in mice. Liver Transpl. 2011 Apr;17(4):418-27.

132. Fanti M, Gramignoli R, Serra M, Cadoni E, Strom SC, Marongiu F. Differentiation of amniotic epithelial cells into various liver cell types and potential therapeutic applications. Placenta. 2017 Mar 30. pii: S0143-4004(17)30220-5.
